# Infiltrating T-cell markers in cervical carcinogenesis: a systematic review and meta-analysis

**DOI:** 10.1038/s41416-020-01184-x

**Published:** 2020-12-01

**Authors:** Tamara R. Litwin, Sarah R. Irvin, Rebecca L. Chornock, Vikrant V. Sahasrabuddhe, Margaret Stanley, Nicolas Wentzensen

**Affiliations:** 1 Clinical Genetics Branch, Division of Cancer Epidemiology and Genetics, National Cancer Institute, Rockville, MD USA; 2 Department of Obstetrics and Gynecology, MedStar Washington Hospital Center, Washington, DC USA; 3 Breast and Gynecologic Cancer Research Group, Division of Cancer Prevention, National Cancer Institute, Rockville, MD USA; 4 Department of Pathology, University of Cambridge, Cambridge, UK

**Keywords:** Adaptive immunity, Tumour immunology, Cervical cancer, Cancer epidemiology, Cancer epidemiology

## Abstract

**Background:**

The host adaptive immune response helps determine which cervical HPV infections persist and progress to precancer and cancer, and systematic characterisation of T-cell infiltration would help inform key steps in cervical carcinogenesis.

**Methods:**

A systematic review and meta-analysis were conducted of infiltrating T-cells in normal cervix, low-grade lesions, high-grade lesions, and invasive cancers including epithelial, stromal, and total tissue and the following markers: CD3, CD4, CD8, FoxP3, CD25, and the CD4:CD8 ratio. An additional qualitative review summarised longitudinal data on associations between infiltrating T-cells and cervical disease persistence, regression, progression, or prognosis.

**Results:**

There were fewer CD3+, CD4+, and CD8+ cells in cervical lesions and more cells in cancers compared to normal epithelium. FoxP3 and CD25+ regulatory T-cell infiltration is high in persistent and precancerous lesions, and longitudinal data show improved outcomes with lower regulatory T-cell levels.

**Conclusions:**

Successful immune evasion may reduce T-cell infiltration in HPV infected and precancerous epithelium, while invasive cancers are highly immunogenic, and regulatory T-cell infiltration increases with cervical disease progression. Understanding these factors may have prognostic value and could aid in novel treatment development and clinical guidelines, but published data are highly heterogeneous and leave important gaps to be filled by future studies.

## Background

Cervical cancer is the most common gynaecologic cancer and has very high burdens of incidence and mortality worldwide, with over 569,000 cases and 311,000 deaths in 2018.^1^ While screening has greatly reduced cervical cancer incidence in high income settings and incidence will be further reduced by vaccination programs, adolescent immunisation will not greatly impact cancer incidence for the next several decades,^2^ and large disparities in incidence are driven by disparities in vaccination and routine screening.

Infection with one of a group of carcinogenic human papillomaviruses (HPV) is the necessary cause of cervical cancer.^3,4^ Over 90 percent of genital HPV infections clear spontaneously within two years,^5^ while those that persist may but do not always progress to precancer.^6^ Even many high-grade lesions spontaneously regress or fail to progress, with evidence suggesting that only 30 percent of cervical intraepithelial neoplasias grade 3 (CIN3) progress to invasive cancer in 30 years after incomplete excision, which may mimic natural history.^7^ While viral factors influence which HPV infections persist and progress, it is hypothesised that the host immune response plays a key role in determining whether an HPV exposure results in infection, persistence, progression, and ultimately carcinogenesis.^8^

Effector T-cells, or T lymphocytes, are a basic component of the adaptive immune response to infectious agents such as viruses that are characterised by expression of the cluster of differentiation (CD) cell surface marker CD3. Major subsets of T-cells include helper T-cells, which express CD4, and cytotoxic or killer T-cells, which destroy infected cells and express CD8. Regulatory T-cells suppress the immune response and commonly express FoxP3 and CD25 in addition to CD4. The adaptive immune response is characterised by complex interactions between these T-cell types and many other components to clear infections without harming uninfected host tissue.

The extent and composition of infiltrating immune cells in cervical tissue affects the natural history of HPV infections because the virus must evade both innate and adaptive immune responses to establish a productive and persistent infection.^9^ HPV infections remain localised in the epithelium and systemic immune response is rare, so circulating inflammatory cells are not typically involved in the immune response to genital HPV infection, and levels of circulating immune markers in response to HPV infection are not easily established.^10–12^ If the innate immune system is unable to prevent an incipient infection, local T-cell responses are necessary to clear the virus, including those mediated by lesion-infiltrating cytotoxic and helper T-cells.^9^

Understanding the composition of T-cell infiltrates in the progression of cervical disease would contribute to etiologic understanding of cervical carcinogenesis as well as revealing possibilities for the development of future clinical interventions. However, despite individual reports of specific markers in population subsets, no collated reference values are available. We, therefore, undertook a systematic review of immunohistochemistry (IHC) and immunofluorescence (IF) literature on infiltrating immune cell counts across cervical disease stages including uninfected normal tissue, HPV infections or low-grade lesions, high-grade lesions or precancers, and invasive cervical squamous cell carcinoma.

## Methods

### Population and measures of interest

The population of interest is women age 18 and above with cervical tissue in one of the following cervical disease states: normal tissue, HPV infected or low-grade lesions, high- grade lesions or precancers, and invasive cervical cancer. The measures of interest include quantified T cell marker prevalence (positive cells per unit area) in epithelial, stromal, and total cervical tissue for the following markers: CD3, CD4, CD8, CD4:CD8 ratio, FoxP3, and CD25.

### Study selection and inclusion criteria

**Fig. 1:**
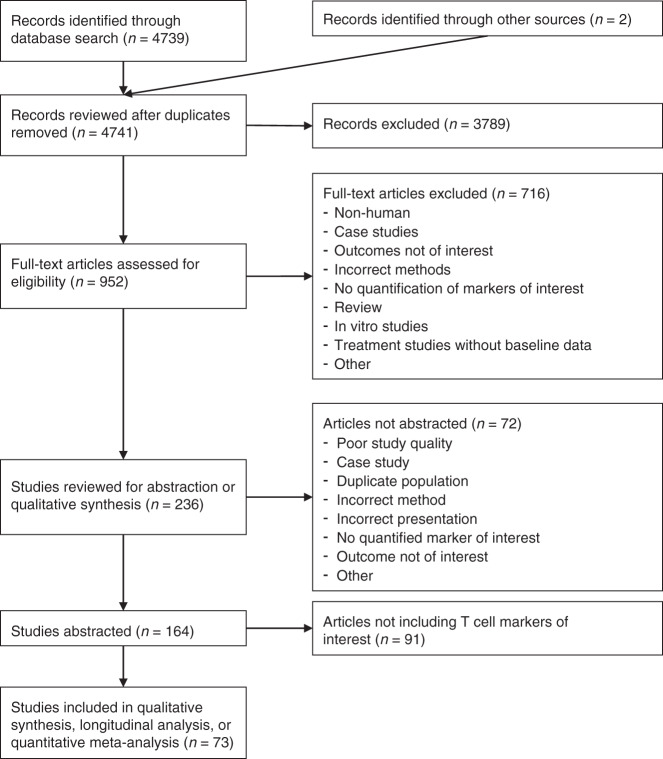
PRISMA diagram. The initial and updated PubMed searches plus two additional records identified during the full-text review resulted in total of 4741 records reviewed. After title and abstract review, 3789 records were excluded leaving 952 for full-text review. The full-text review excluded an additional 716 records, leaving 236 records for potential abstraction. Of these, 72 were not abstracted for the reasons indicated and 164 were abstracted. After limiting to the T-cell markers of interest, 73 records remained for inclusion in the quantitative meta-analysis, longitudinal analysis, or qualitative summary of infiltrating T-cells in cervical tissue.

The systematic review and meta-analysis were performed using the preferred reporting items for systematic reviews and meta-analyses (PRISMA) structure for the conduct of systematic reviews (Fig. 1). The search algorithm to identify peer-reviewed articles in the PubMed database that quantified pre-specified immune cell types was as follows:

“((cellular immune response) OR lymphocytes OR macrophages OR APC OR (natural killer cells) OR monocytes OR (toll-like receptors) OR t-cells OR CD11C OR CD33 OR CD23 OR CD37 OR CD53 OR CD63 OR CD81 OR CD57 OR CD163 OR CD195 OR EMR1 OR IL-22 OR CD25 OR CTLA4 OR GITR OR CD62L OR CCR7 OR CD44 OR CD127 OR STAT5 OR CCR4 OR APMs OR CD7 OR CD3 OR CD4 OR CD8 OR cytokines OR CD68 OR CD56 OR FOXP3 OR CD14 OR CD16 OR CXCR3 OR TLR OR IL-6 or S100) AND ((cervix) OR (cervical cancer) OR (CIN) OR (cervical intraepithelial neoplasia) OR (cervical precancer) OR (cervical precancerous lesions) OR (human papillomavirus) OR (HPV)) AND (IHC OR immunohistochemistry OR immunohistochemical OR histology OR histologic OR FFPE OR (formalin-fixed paraffin embedded) OR (formalin-fixed paraffin embedded) OR (formalin-fixed paraffin-embedded) OR (formalin-fixed paraffin-embedded) OR infiltrate OR infiltrating OR infiltration).”

The search was conducted through September 5th, 2019. Eligible studies included original cross-sectional, cohort, and case-control studies that used IHC or IF methods to identify T-cell populations in cervical tissues, including biopsies, hysterectomy specimens, or tissue microarray samples. Exclusion criteria included studies of non-human animals, studies conducted in vitro, studies not examining outcomes of interest (cervical cancer, pre-cancer or histologic changes of the cervix), other methods (studies conducted with blood, serum or mucosa samples, via flow cytometry, or fine needle aspiration), case studies, studies lacking quantified immune markers of interest, treatment or prevention studies without baseline data, studies among pregnant women, and records that were not original research articles. Where multiple studies published duplicative or incremental results, including data from the same women, only one was used. In order to focus on the adaptive T-cell immune response, the marker set of interest was limited to CD3, CD4, CD8, the CD4:CD8 ratio, FoxP3, and CD25. A search of the EMBASE database was conducted using the same search terms and yielded no additional papers eligible for inclusion.

An initial set of 150 titles and abstracts were reviewed separately by three reviewers (SI, TL, and NW) to reach consensus about inclusion standards. The remaining records underwent a title review by SI or TL for potential inclusion, and reviewers switched manuscript sets to complete abstract reviews. Full-texts were evaluated by both investigators separately, and reference lists of full-text papers were checked for additional relevant references. Discrepancies between the reviewers for individual studies were resolved by consensus.

### Data abstraction and outcome characterisation

The primary outcome was mean positively stained cells per unit area of sections of uterine cervical tissue. Where means were not available, medians with interquartile ranges (IQR) or minimum and maximum counts were abstracted. Data from figures not numerically reported in text were abstracted by estimating counts from data points and error bars or box plot bounds. Conditional cell counts were not abstracted except for the ratio of interest CD4:CD8 and the ratios CD25:CD4 and FoxP3:CD4 under the assumption that CD25- and FoxP3-expressing regulatory T-cells are subsets of CD4-expressing T-cells.^13^ We also conducted a quality review evaluating the likelihood of confounding, selection bias, and information bias with the signalling questions suggested for systematic reviews of observational studies of epidemiology (COSMOS-E) placed in the framework of quality assessment tool for diagnostic accuracy studies-2 (QUADAS-2).^14,15^ Due to the descriptive nature of the reports, the risk of reporting bias was considered low both within and between studies and was not used for data synthesis.

### Outcome standardisation

Outcome measures were standardised to mean and SD in cells/mm^2^ in preparation for meta-analysis. Measures reported in units of high power fields (HPF) with 400x magnification were converted to cells/mm^2^ by multiplying by a factor of 5.10 assuming an ocular field number of 20 and the equivalent area of 1 HPF of 0.196 mm^2^.^16,17^ If the magnification was not specified, studies were excluded. Median and IQR were converted to mean and SD according to the method of Wan et al.^18^ Studies reporting results in units of cells per unit area other than square millimetres were multiplied by the appropriate conversion factor. If SD was not reported but a hypothesis test was performed with a test statistic or p-value reported, SD was derived from the appropriate test statistic formula to obtain study-specific estimates. In studies reporting a mean and range, SD was computed as described in Wan et al.^18^ Finally, simple imputation procedures were used to obtain remaining missing SD values using gamma distribution models within strata of marker, disease classification and tissue type with at least two independent studies with SD values reported.

In addition to the primary outcome, we abstracted the following variables stratified by disease classification and immune marker where available: age, HIV status, HPV status, tissue site (transformation zone, ectocervix, or endocervix), tissue type (epithelial, stromal or total), country where the study was conducted, and cancer stage and type (squamous or adenocarcinoma). Country of origin, HPV status, and age were not considered in the final analyses. Papers that did not designate epithelial or stromal tissue were assumed to report values from entire slides or cores consisting of both tissue types (total), and meta-analyses were conducted separately for each of the three type designations. The local immune environment and risk of cervical disease are greatly altered in immunocompromised individuals, so studies of HIV positive women were excluded from the present analysis.

### Meta-analysis

Random-effects meta-analyses produced pooled estimates of mean cells/mm^2^ and 95% confidence intervals for each marker by tissue type within each disease level classification. Prioritisation was in the following sequential order for samples taken from multiple cervical locations in the same women: (1) transformation zone (2) ectocervix (3) endocervix. ANOVA and Kruskal–Wallis tests were used to evaluate differences across disease level means.

Analyses for CD3+, CD8+, CD4+ and the CD4:CD8 ratio incorporated both explicitly reported and calculated values. Under the assumption that CD3 positive T-cells are also positive for either CD4 or CD8,^19^ means and SDs for any of these markers can be derived from the values of the other two markers. A reported value for the CD4:CD8 ratio is also informative under this assumption if the absolute mean for at least one of CD3, CD4, or CD8 is also reported, so means and SDs for the remaining markers were calculated within populations that reported at least two of the following: CD3, CD4, CD8, and the CD4:CD8 ratio.

### Definitions and classifications of cervical disease

Cervical disease classification terminology and diagnoses are complex and have evolved throughout the published literature.^20^ All historical and modern cervical disease classification were included in the systematic review criteria, including normal/non-dysplastic, cervical intra-epithelial neoplasia (CIN) grades 1-3, low-grade CIN (lgCIN), high-grade CIN (hgCIN), low-grade and high-grade squamous intraepithelial lesion (LSIL and HSIL), and microinvasive or invasive squamous cell carcinoma or adenocarcinoma with or without lymph node involvement. Carcinomas in situ and neuroendocrine or other rare tumours were excluded, as were genital warts (condylomas) and cervicitis.

Four disease classifications were evaluated in the primary analysis: normal, low-grade neoplasia, high-grade neoplasia, and cancer. Normal samples had no evidence of neoplasia and were taken from healthy controls, hysterectomies for benign uterine diseases, or screening populations. Samples reported as CIN1, LSIL, and lgCIN were grouped as low-grade cervical neoplasia for analysis, and CIN2, CIN3, HSIL and hgCIN were grouped as high-grade cervical neoplasia. Because in general 90 percent of invasive cervical cancers are squamous cell carcinomas (SCC) and the immune infiltrates within the tissues of origin (ectocervix or transformation zone for adenocarcinomas, endocervix for squamous cancers) differ, we limited the quantitative meta-analysis to studies reporting only SCC or unspecified invasive cervical carcinomas.^21^ Studies of only adenocarcinomas were excluded.

### Sensitivity analyses

Sensitivity analyses were performed to confirm that the data abstraction and imputation processes did not substantially alter the results. These included stratified analysis by study unit designation (HPF versus cells per unit area), analysis of CD3, CD4, CD8 and CD4:CD8 with only explicitly reported (non-derived) data, including only the subset of cancer observations explicitly reported as squamous cancer and excluding cancers of unspecified subtype, incorporating all cancer observations including both squamous and adenocarcinomas in the cancer disease classification, and excluding reports of cancer- or lesion-adjacent normal tissue out of concern that these samples may not represent a truly normal immune microenvironment.

Analyses were completed using SAS version 9.4 (SAS Institute, Cary NC) and R version 3.6.1 run on R Studio version 1.1.^22,23^ Meta-analyses were completed and forest plots were generated using the “meta” package version 4.9-5^24^ and GraphPad Prism 8.

## Results

### Systematic review

The PubMed search identified 4739 records, and two additional records were identified in references of other records (Fig. 1). Title, abstract, and full-text review yielded 236 manuscripts for potential abstraction. At the abstraction stage, additional exclusion criteria included duplicate populations and inadequate descriptions of methods and results for abstraction of quantified marker data, resulting in 72 additional manuscripts excluded. A total of 73 studies reported results of interest, including 61 studies with quantitative counts of T-cell markers reported in a manner suitable for meta-analysis (Table S1),^25–85^ 9 studies reporting CD25 levels which were qualitatively summarised, and 20 studies reporting longitudinal data (some studies were included in multiple categories). A quality review of all studies included in the manuscript revealed many studies with the uncertain likelihood of bias or confounding due to insufficiently detailed reporting of patient populations and methods (Table S2). In line with COSMOS-E guidelines, no studies were excluded due to results of the quality review.^14^ HPV status was not reported in studies of normal tissues (women without active HPV infections) and ubiquitous HPV positivity can be assumed for all other disease levels, so this variable was not included in the meta-analyses.

### Results summary

**Fig. 2:**
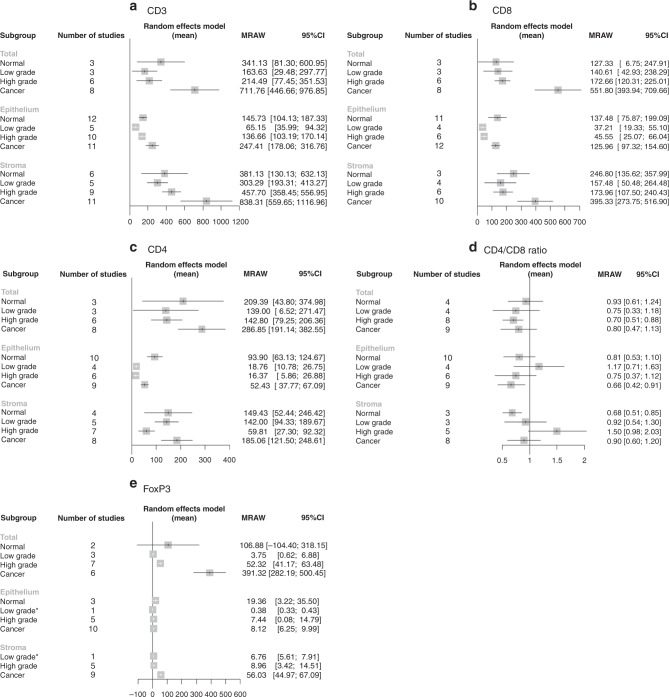
Forest plots of T-cell infiltrate quantitative meta-analyses. Forest plots of meta-analyses of infiltrating T-cell markers across cervical disease stages in total tissue, epithelium and stroma expressed as mean cells/mm^2^ with 95% confidence intervals for the following markers: **a** CD3, **b** CD8, **c** CD4, **d** CD4:CD8 ratio, **e** FoxP3. There were no stromal normal FoxP3 reports, so this category is absent. CIN cervical intraepithelial neoplasia, LG low grade, HG high grade, MRAW raw (untransformed) mean, CI confidence interval.

A formal meta-analysis was conducted of CD3 (pan T-cell), CD8 (cytotoxic T-cell), and CD4 (helper T-cell) counts at each of four disease levels (normal, lgCIN, hgCIN, and invasive cancer) in each of three tissue sections (total, epithelium, and stroma) according to how results from each study were reported in the manuscripts (Figs. 2 and S1). ANOVA and Kruskal–Wallis tests showed statistically significant differences between disease levels only for CD8 total tissue (*p*-values 0.016 and 0.011, respectively, Figure S2), with the group differences driven by significant pairwise differences between cancer and each other disease stage (*p*-values 0.008 vs hgCIN, 0.049 vs lgCIN, and 0.024 vs normal tissues). Heterogeneity (*I*^2^) between studies was high for all markers (Fig. 2).

### Pan T-cells (CD3+)

The overall pattern of CD3+ cells was the same in all three tissue sections, with the lowest numbers of T-cells present in lgCIN and hgCIN, higher numbers in normal cells, and the highest numbers in cancer (Figs. 2a and S1A). In total tissue, normal tissue had a mean of 341 cells/mm^2^ (95% CI 81–601); lgCIN had 164 (29–298); hgCIN had 214 (77–352); and cancer had 712 (447–977). In epithelium, normal tissue had a mean of 146 cells/mm^2^ (104–187); lgCIN had 65 (36–94); hgCIN had 137 (103–170); and cancer had 247 (178–317). Stromal tissue adjacent to normal epithelium had a mean of 381 cells/mm^2^ (130–632); lgCIN was 303 (193–413); hgCIN was 458 (358–557); and cancer was 838 (560–1117).

### Killer T-cells (CD8+)

CD8+ cell patterns in epithelial and lesion-adjacent stromal tissue were mostly similar to those for overall CD3+ T-cell count, with higher infiltration in normal and tumour epithelial and adjacent stromal tissue and lower infiltration in lgCIN and hgCIN (Figs. 2b and S1B). In epithelium, normal tissue had a mean of 137 cells/mm^2^ (95% CI 76–199); lgCIN had 37 (19–55); hgCIN had 46 (25–66); and cancer had 126 (97–155). In stroma, normal tissue had a mean of 247 cells/mm^2^ (136–358); lgCIN had 157 (50–264); hgCIN had 174 (108–240); and cancer had 395 (274–517).

### Helper T-cells (CD4+)

The meta-analysis of CD4+ helper T-cells also revealed higher infiltration in normal and cancer tissue and lower infiltration in lgCIN and hgCIN tissue, most notably in hgCIN (Figs. 2c and S1C). In total tissue, normal tissue had a mean of 209 cells/mm^2^ (95% CI 44–375); lgCIN had 139 (7–271); hgCIN had 143 (79–206); and cancer had 287 (191–383). In epithelium, normal tissue had a mean of 94 cells/mm^2^ (63–125); lgCIN had 19 (11–27); hgCIN had 16 (6–27) and cancer had 52 (38–67). In stroma, normal tissue had a mean of 149 cells/mm^2^ (52–246); lgCIN had 142 (94–190); hgCIN had 60 (27–92); and cancer had 185 (122–249).

### CD4/CD8 ratio

CD4:CD8 ratios varied widely across cervical disease stages. A few disease stage-tissue type combinations revealed higher CD8 than CD4 levels (normal stroma, hgCIN total tissue, and cancerous epithelium), but most confidence intervals included the null value of one (Figs. 2D and S1D). There was no significant decrease in the CD4:CD8 ratio in tumour tissue, although there is a suggestion of such a decrease in epithelium (intratumoural tissue).

### Regulatory T-cells (FoxP3+ and CD25+)

There was no clear pattern to the number of FoxP3+ regulatory cells/mm^2^ at each disease level in epithelial tissue and high heterogeneity in total tissue, while there was an increase across lgCIN, hgCIN, and cancer in lesion-adjacent stromal tissue with no studies reporting FoxP3 levels in normal stroma (Figs. 2E and S1E). Some tissue type-disease level combinations had a single report, which was reproduced in the figure without pooling for meta-analysis. In total tissue, normal tissue had a mean of 107 cells/mm^2^ (95% CI -104–318); lgCIN had 4 (1–7); hgCIN had 52 (41–63); and cancer had 391 (282–500). In epithelium, normal tissue had a mean of 19 cells/mm^2^ (3–36); lgCIN had 0.4 (0.3–0.4); hgCIN had 7 (0–15); and cancer had 8 (6–10). In stroma, normal tissue had no studies reporting, while lgCIN had a mean of 7 cells/mm^2^ (6–8); hgCIN had 9 (3–15); and cancer had 56 (45–67). FoxP3 interpretation was limited by low numbers and low quality of studies in many of the categories including lgCIN epithelium and stroma in a single study each and no manuscripts reporting normal stromal tissue. One manuscript which was not suitable for meta-analysis reported a significant trend towards increasing FoxP3 infiltration with increasing severity of cervical disease.^86^

CD25 data were not formally meta-analysed due to insufficient study numbers (Table S3). However, several independent reports suggested that CD25 positive cell levels and the proportion of CD4+ or CD8+ cells which were also CD25+ increased with increasing severity of cervical disease in both epithelial and lesion-adjacent stromal tissue.^26,87^ Additionally, there were reports of increasing FoxP3 as a proportion of CD4+ or CD8+ cells with increased disease severity as well as confirmation that FoxP3 and CD25 are generally co-expressed and may therefore be used interchangeably as regulatory T cell indicators.^26,62,88^ Consistent with the idea that an increased regulatory T-cell population is associated with worse disease, there was also a report of greater regulatory T-cell-associated cytokine profile in hgCIN compared to lgCIN^68^ and a report of an association between lower CD25 levels and lower CD25:CD4 and CD25:CD8 ratios and the regression of hgCIN.^67,89^ Several studies suggested that the regulatory T-cell (FoxP3+ and/or CD25+) presence was greater in stroma or peritumoural tissue than in the epithelium or intratumoural tissue, especially as carcinogenesis progressed.^26,30,52,67,88,89^ Finally, there were also several reports that in hgCIN lesions, HPV16 infections are associated with higher CD25+ infiltration levels than other HPV types.^67,87,89^

### Sensitivity analyses

Several sensitivity analyses were conducted to determine if results were robust to various analytical decisions. These included stratifying the meta-analysis into reports of cells/unit area and reports of cells/HPF; restricting the analysis to explicitly measured data for CD3, CD4, CD8, and the CD4:CD8 ratio rather than including imputed values for these markers; excluding all not-explicitly squamous cancers; including all cancers including adenocarcinomas; and excluding reports included cancer- or lesion-adjacent tissue from the normal tissue analysis. The observed patterns of immune cells infiltration were generally robust to sensitivity checks, though there were losses of clarity and increases in confidence intervals as study numbers decreased in many categories, sometimes to no studies or a single study, making interpretation challenging (Table S4).

### Longitudinal analysis of T-cell markers

In addition to overall quantification of T-cell markers, 20 references included relevant longitudinal or follow-up data on the effect of infiltrating T-cell markers on regression, progression, recurrence or patient survival (Table 1). Among studies that followed women with CIN lesions for regression, persistence, progression, or recurrence after treatment, several reported no association between CD3 pan T-cell count and clinical course of CIN lesions, while one study associated high CD3 with lesion recurrence after conisation.^59,81,90,91^ For CD8+ cells, five studies reported no significant association with regression, progression, persistence, or recurrence.^59,66,81,90,91^ Four studies reported an association between high CD8 infiltration and CIN lesion regression: two used statistical tests to compare CD8+ quantities in persistent infections to those that regressed,^89,92^ one modelled the hazard of regression for those with high vs low CD8+ expression,^67^ and one reported a suggestive association between highly active infiltrating CD8+ T-cells and lesion regression.^85^ For CD4+ helper T-cells, one study reported that lower CD4 levels are associated with recurrence, while five studies showed no association.^66,81,85,90,91^ A reduced CD4:CD8 ratio was associated with both increased CIN lesion regression and decreased progression, although one study showed no association.^81,90,91^ For regulatory T-cells, two manuscripts analysing the same dataset found lower CD25 and lower CD25:CD4 and CD25:CD8 ratios associated with regression in hgCIN,^67,89^ while one study reported no association between FoxP3 levels and lgCIN regression.^85^ In sum, reports of infiltrating T-cells on CIN natural history are mixed but generally suggest that greater numbers of (non-regulatory) T-cells are observed with improved outcomes.

**Table 1:** Records including longitudinal data.

				T-cell markers						Age		Method
First author	Year	Outcome	Disease population	CD3	CD4	CD8	CD4:CD8	FoxP3	Other	Years	Reporting metric	IHC/IF
Ancuta	2014	Relapse	Cancer	X						NR		IHC
Bethwaite	1996	Survival	Cancer	X						43.7	Mean	IHC
Edwards	1995	Recurrence	Neoplasia		X	X				NR*	NR	IHC
Enwere	2017	Survival	Cancer			X				44	Median	IHC
Grochot	2019	Survival	Cancer					X		44	Median	IHC
Hellberg	2009	Survival	Cancer			X				59.7	Mean	IHC
Jordanova	2008	Survival	Cancer					X	CD8:FoxP3	48.5	Mean	IF (CD8); IHC (FoxP3)
Karageorgopoulou	2017	Survival	Cancer		X	X				58	Median	IHC
Maluf	2008	Recurrence	CIN3	X		X			CD68, CD45RO	34.9	Mean	IHC
Nedergaard	2008	Relapse	Cancer	X	X	X	X		CD57, CD68, CD45RO, CD45RA	41.5	Mean	IHC
Origoni	2013	Recurrence	hgCIN		X	X				37	Mean	IHC
Ovestad	2011	Regression	hgCIN			X			CD25, CD4:CD25, CD8:CD25	NR	NR	IHC
Punt	2015	Survival	Cancer	X				X		40	Median	IF
Saglam	2019	Survival	Cancer			X				47	Mean	IHC
Shah	2011	Survival	Cancer		X	X	X	X		47	Median	IHC
Syrjanen	1985	Regression/Persistence/Progression	Normal, CIN	X	X	X	X			28.6	Mean	IHC
Syrjanen	1987	Regression/Persistence/Progression	Normal CIN	X	X	X	X			28.7	Mean	IHC
Trimble	2010	Regression	hgCIN			X				NR	NR	IHC
Vayrynen	1985	Regression/Persistence/Progression	CIN	X	X	X	X			25-29	Median	IHC
Woo	2008	Regression/Progression	CIN		X	X		X	CD56	20-30	Range	IHC

A total of 20 records were identified in the systematic review as containing longitudinal follow-up data on infiltrating T-cells makers and cervical disease outcome. The range of markers is indicated, as is the initial diagnosis (HPV infection, CIN/neoplasia, or cancer), what outcomes were followed, patient age, and method used.

*CIN* cervical intraepithelial neoplasia, *FoxP3* forkhead/winged-helix transcription factor box P3, *NR* not reported, IHC immunohistochemistry, *IF* immunofluorescence.

*Cancer patients average 15 years older than CIN patients.

Among analyses of cancer relapse or survival, most studies detected significant improvements in prognosis with increased infiltrating T-cell populations. Intratumoural or peritumoural CD3+ cells were associated with increased overall survival and disease-free survival, lower rates of relapse, earlier stage disease and smaller tumour size at diagnosis, and reduced lymph node spread.^32,63,73,93,94^ One study reported that higher CD3+ infiltration was associated with disease-free survival in adenocarcinoma patients.^73^ The picture for CD8+ was more mixed, with one study showing a significant association between increased CD8 and lower overall survival, one showing association with decreased risk of relapse, one study showing an association with improved overall survival (for both intratumoural/epithelial and peritumoural(lesion-adjacent)/stromal CD8) in early stage cancer only, and other studies suggesting no association with overall or progression-free survival.^40,77,94–96^ For CD4, two studies showed an association between infiltrate density and reduced relapse or increased survival, while one showed no association with overall or progression-free survival and a fourth detected an association only for late stage disease.^77,94,95,97^ For the CD4:CD8 ratio, there is one report of an increased ratio associated with 5-year survival and one of no association.^77,94^ For regulatory T-cells, two studies reported that infiltrating FoxP3+ cells are associated with decreased survival, one reported no association and one adenocarcinoma study linked FoxP3 with improved survival.^51,73,77,98^ Overall, the literature mostly reports that infiltrating CD3 and CD4 T-cells have a positive effect on cervical cancer prognosis, while regulatory T-cells may have a negative effect.^99^

## Discussion

**Fig. 3:**
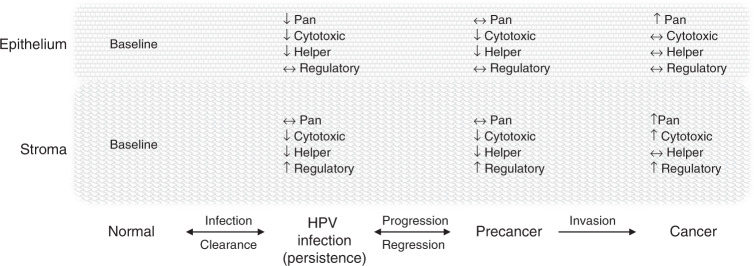
Conceptual model of infiltrating T-cells in cervical carcinogenesis. Normal cervical tissue is infiltrated by T-cells and T-cell subsets that respond to infection as part of the adaptive immune response. When HPV infection becomes established and is not immunologically cleared, it evades immune detection with reduced cytotoxic (CD8+) and helper (CD4+) T-cell infiltration in both the epithelium and adjacent stromal tissue. It is unclear whether regulatory (CD25+, FoxP3+) T-cell infiltration is affected but some evidence suggests it begins to increase. When HPV infections persist and progress to cervical precancer, pro-immune T-cell subsets continue to be suppressed and regulatory (inhibitory T-cells) amplified. If the lesion progresses to invasive cancer, a highly immunogenic state is reached with high levels of pan, cytotoxic, and helper T-cells in the surrounding stroma and to some extent in the tumour itself. Regulatory T-cells may be relatively high, resulting in a worse prognosis, or low, resulting in a better prognosis.

A systematic review and meta-analysis were undertaken to establish how infiltrating T-cell populations vary across cervical disease stages. Studies of infiltrating CD3, CD4, CD8, FoxP3, or CD25 positive cells and the CD4:CD8 ratio in the epithelium, stroma, or total tissue of normal, lgCIN, hgCIN, or cancer were abstracted for inclusion in the review. The results suggest a pattern of higher T-cell infiltration in normal and especially in cancerous cervical tissue and lower infiltration in low- and high-grade CIN for CD3, CD4, and CD8 across tissue types, with a few deviations (Fig. 3).

CD3 is a pan-T-cell marker, incorporating many subsets of T-cells that express and release various receptors and cytokines.^100^ Infiltrating CD3 levels in cervical lesions and tumours may give an indication of general immune activity as the cervical disease progresses. The reduction in infiltrating T-cells in cervical lesions relative to normal cervix supports that evasion of the adaptive immune system by active and transformed HPV infections is central to the development of cervical cancer. The absolute numbers of infiltrating CD3+ cells reported were higher in stromal than in epithelial tissue across disease stages, which suggests that T cell responses may be more robust in or may originate in the stromal tissue.^101^

Cells that express CD8 are commonly characterised as cytotoxic or killer T-cells that destroy infected or damaged cells, although the assumption that CD8+ cells signify active adaptive immunity in cervical lesions remains unproven. Assuming CD8+  cells function as an integral component of adaptive immunity, it is expected that an increased presence of these cells would be detected at the site of a tumour or active infection. However, as for pan T-cells, the finding that cytotoxic T-cell counts are reduced in CIN lesions fits with the overall picture of immune evasion as characteristic of persistent HPV infections and cervical lesions. The pattern for CD8 was not as consistent across tissue types as it was for CD3 and was particularly difficult to interpret for total tissue because of the wide confidence intervals for each disease level.

Helper T-cells activate cytotoxic T-cells and are characterised by expression of CD4, although there are also subsets of CD4+ cells with other functions such as regulatory T-cells. The pattern of CD4+ cell infiltration was similar to that of CD3, with reduced infiltrate in both low- and high-grade lesions relative to normal tissue, and the greatest infiltration in cancer. It has been reported that T-cell responses to HPV E2, a marker of productive infection, are associated with regression, while T-cell responses to HPV E6, a marker of transforming infection, are associated with persistence.^102^ However, T-cell specificity cannot be detected using conventional immunohistochemical techniques.

Regulatory T-cells inhibit the immune response and may therefore promote carcinogenesis, in contrast to T-cells overall which are associated with suppression of carcinogenesis. Indeed, a previous analysis, albeit of only two studies, concluded that high levels of FoxP3+ cells are associated with significantly reduced overall survival in cervical cancer.^103^ Our findings in the FoxP3 meta-analysis and CD25 qualitative summary also suggest that an increased presence of regulatory T-cells is associated with more severe disease, that regulatory T-cell infiltration may inhibit the immune response at all stages of cervical carcinogenesis, and that this effect may be greater for HPV16, the most highly carcinogenic HPV type. Further tissue analysis is required to confirm this hypothesis.

The results were generally robust to sensitivity analyses, which had small effects on absolute estimates overall. Stratifying by data reporting method greatly reduced the number of studies in each category, rendering the results uninterpretable. Restriction to explicitly reported values for CD3, CD4, CD8, and the CD4:CD8 ratio likewise reduced the precision of the estimates, but the overall patterns generally remained. Including only explicitly squamous cancers and including all cancers including explicitly adenocarcinomas had little effect because close to 90% of all cervical cancers are squamous cell carcinomas, and possibly because T-cell infiltrates increase in adenocarcinomas similarly to the patterns in squamous cell carcinomas. Only two studies used cancer-adjacent normal tissue, so removing them also had only small effects on the estimates and no effect on the interpretation.

In addition to cross-sectional analyses, longitudinal data in both CIN lesions and cancer suggest that increased overall and helper T-cell subsets are associated with improved prognosis, a finding that has also been observed in HPV positive vulvar squamous cell carcinoma.^104^ Many studies showed no association, perhaps due to lack of power, seven showed an association of greater numbers of non-regulatory T cells or subsets with better outcomes, and just one reported an association of greater T cells with worse outcomes. As expected, the opposite is true of regulatory T cells, with both studies that reported significant findings associating lower levels of regulatory T cells with better outcomes. These findings support models of HPV-associated carcinogenesis that suggest immune evasion and suppression are critical to the carcinogenesis process, but that once an invasive cancer is established it is highly immunogenic. This is also consistent with previous models of cellular immune response in cervical disease progression.^101,105^

### Limitations

The data on infiltrating immune cells in cervical tissue are highly heterogeneous. Patient populations, laboratory techniques, environmental exposures, and other factors may vary between studies, and some studies report estimates from only the most highly infiltrated parts of slides while others review randomly selected sections. Many studies reported only semi-quantitative results, such as binary or categorical classifications of immune infiltrates. While we were able to include studies reporting cells/HPF that also reported the microscope settings necessary to convert to an area-based measurement, not all studies included such information, and in some cases, it was necessary to impute or exclude study results. The publication dates of abstracted manuscripts range from 1985 to 2018, and improvements in antibody quality and IHC technique over this 30-year span may reduce the comparability of results over time. In particular, the FoxP3 results must be qualified by the understanding that there may not be good agreement between different FoxP3 antibodies in the early literature, so absolute FoxP3 counts, in particular, may not be reliable.^106^ There is a clear need for greater standardisation of reporting of tissue analysis results to improve the ability to pool data across studies, as lack of standardisation limited our ability to draw clear conclusions from the data. Automated image analysis could also help improve comparability between datasets. In addition, very few studies reported HPV type, which affects HPV persistence and progression to cervical precancer and cancer.^107^ The composition of lymphocytes has also been reported to vary by location within the cervix (transformation zone, ectocervix, endocervix),^108^ but most studies did not report this variable, so the current analysis does not take this factor into account other than to include the transformation zone in the analysis where multiple samples were taken from the same woman because it is the most common site of squamous cancer development. The present analysis also cannot address the reasons for differential adaptive immune responses to HPV infection between individuals, which likely include both environmental factors such as coinfections with other sexually transmitted infections and genetic factors such as HLA alleles.^109^

### Studies of blood

Some previous studies have been undertaken in peripheral blood, for instance it has been reported that CD25+ regulatory T-cell levels, which can inhibit cytotoxic and helper T-cell responses, are elevated in blood in patients with severe cervical lesions and cancer.^11,110^ There is also evidence that the immune response is deregulated during cervical carcinogenesis^11,111,112^ and that the immunosuppressive environment generated by HPV is a critical factor in disease progression.^113^ It has also been reported that general immune response to challenges other than HPV itself may be reduced in women with persistent HPV infections, with a greater effect in older women, although the direction of any causal relationship between suppressed immune function and HPV persistence is unclear.^114^ However, in general, studies in peripheral blood are not reflective of the local immune marker population at the site of HPV infection,^115^ and it is important that studies be conducted in tissue given the localised nature of the immune response to HPV infection.

### Future work

Further primary tissue analyses are clearly needed to clarify the role of T-cell infiltrates in cervical disease. The current analysis excluded immune infiltrates other than T-cells such as Langerhans cell markers S100 and CD1a that also likely play a role in the immune response to HPV infection and cervical carcinogenesis. In addition, new technologies such as multiplex immunofluorescence and RNAscope could be applied to archival tissue to characterise immune infiltrates and other features such as gene expression in much greater detail.^116^ Studies of HIV positive individuals were also excluded due to known differences in HPV-associated carcinogenesis in immune-suppressed populations. Future analyses of these additional markers and populations may shed further light on the role of the adaptive immune system in control of HPV infection. In addition to further etiologic understanding of cervical carcinogenesis, improved knowledge of patterns of T-cell infiltration across cervical carcinogenesis may inform the development of therapeutic vaccines to induce lesion regression by inducing a response to viral antigens E6 and E7, both of which are markers of transforming HPV infection.^117^ It could also provide insight into the use of immune checkpoint inhibitors or other immunotherapies in cervical cancer patients.^118^

## Supplementary information

Supplemental Data

## Data Availability

All data analysed in the study are available in the cited research articles; no new data were generated.
