# Supplementary material for: Infiltrating T-cell markers in cervical carcinogenesis: a systematic review and meta-analysis
Source: Br J Cancer. 2020 Dec 1;124(4):831–41. doi: 10.1038/s41416-020-01184-x (PMC7884592; doi:10.1038/s41416-020-01184-x)
Supplement: Supplementary file 1 — Supplemental Data [file 41416_2020_1184_MOESM1_ESM.pdf]

Figure S1. Full Forest Plots

CD3						
**Subgroup**	**Number of studies**	**Sample Size**	**Study authors**	**Random effects model**	**Mean**	**95% CI**
**Total**						
Normal	3	13	Edwards 1995		157.08	118.72 – 195.44
		17	Bell 2000		861.90	761.82 – 961.98
		37	Adurthi 2008		28.00	23.87 – 32.13
			Pooled		**341.13**	**81.30 – 600.95**
Low grade	3	16	Edwards 1995		349.86	296.97 – 402.75
		14	Bontkes 1997		77.88	59.42 – 96.34
		15	Jaafar 2009		69.70	33.38 – 106.02
			Pooled		**163.63**	**29.48 – 297.77**
High grade	6	20	Edwards 1995		474.30	411.23 – 537.37
		7	Bontkes 1997		147.17	123.12 – 171.22
		10	Bontkes 1997		101.08	65.81 – 136.35
		30	Adurthi 2008		441.00	423.73 – 458.27
		15	Jaafar 2009		68.00	50.79 – 85.21
		20	Jaafar 2009		62.90	57.93 – 67.87
			Pooled		**214.49**	**77.45 – 351.53**
Cancer	8	16	Edwards 1995		783.36	691.77 – 874.95
		14	Bontkes 1997		163.13	115.19 – 211.07
		22	Hachisuga 2001		1354.05	1092.50 – 1615.60
		13	Hachisuga 2001		1281.63	925.73 – 1637.53
		30	Adurthi 2008		699.00	685.30 – 712.70
		7	Jaafar 2009		142.80	78.43 – 207.17
		15	Jaafar 2009		125.80	68.45 – 183.15
		67	Wang 2014		1344.16	1188.83 – 1499.48
			Pooled		**744.46**	**446.66 – 976.85**

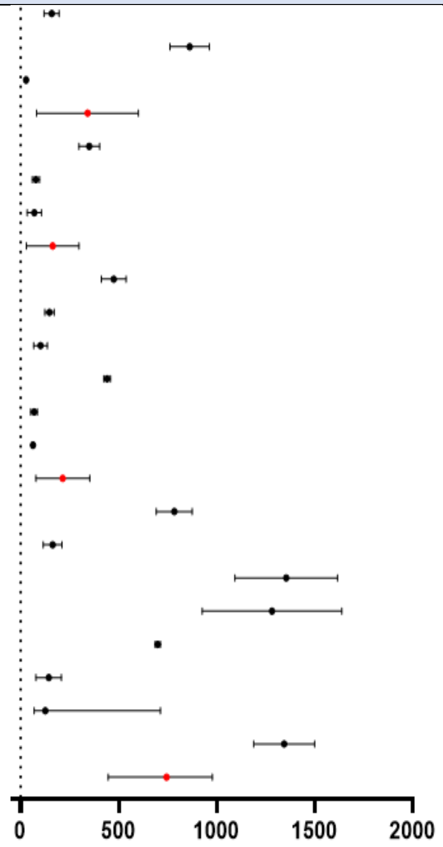

Epithelial

Normal	12	29	Poppe 1995	565.00	437.27 – 692.73
		9	Poppe 1995	438.67	188.72 – 688.61
		5	Rosini 1996	106.60	68.30 – 144.90
		150	Szarweski 2001	233.67	219.56 – 247.77
		11	Kobayashi 2004	470.00	137.30 – 802.30
		16	Pudney 2005	315.00	295.87 – 334.13
		4	Monnier-Benoit 2006	94.67	61.56 – 127.77
		9	Nedergaard 2007	115.00	90.32 - 139.68
		115	Nedergaard 2007	30.71	28.78 – 32.65
		9	Jaafar 2009	22.10	17.16 – 27.04
		15	Jaafar 2009	35.70	24.23 – 47.17
		7	Carrero 2015	35.70	13.03 – 58.37
			Pooled	**145.70**	**104.13 – 187.33**
Low grade	5	19	Rosini 1996	84.20	65.45 – 102.95
		9	Monnier-Benoit 2006	62.00	47.47 – 76.53
		5	Monnier-Benoit 2006	89.67	76.28 – 103.05
		26	Bedoya 2013	25.75	20.30 – 31.20
		45	Carrero 2015	66.30	52.89 – 79.71
			Pooled	**65.15**	**35.99 – 94.32**
High grade	10	16	Coleman 1994	371.00	315.88 – 426.12
		19	Rosini 1996	149.50	112.76 – 186.24
		14	Kobayashi 2004	601.00	412.89 – 789.11
		13	Monnier-Benoit 2006	63.33	55.91 – 70.76
		59	Woo 2008	54.52	45.22 – 63.82
		10	Loddenkemper 2009	195.50	145.23 – 245.77
		21	Bedoya 2013	17.15	8.77 – 25.53
		25	Bedoya 2013	23.50	14.67 – 32.33
		10	Carrero 2015	127.50	99.05 – 155.95
		10	Carrero 2015	204.00	172.39 – 235.61
			Pooled	**136.66**	**103.19 – 170.14**
Cancer	10	7	Rosini 1996	133.10	89.24 – 176.96
		49	Bethwaite 1996	386.00	354.00 – 418.00
		11	Monnier-Benoit 2006	161.00	90.80 – 231.20
		102	Needergaard 2007	684.33	565.88 – 802.79
		20	Needergaard 2007	1334.33	973.99 – 1694.68
		115	Jordanova 2008	83.24	70.82 – 95.67
		12	Loddenkemper 2009	263.50	122.74 – 404.26
		10	Shah 2011	235.93	158.72 – 313.13
		30	Shah 2011	281.11	219.76 – 342.47
		24	Bedoya 2013	20.94	16.00 – 25.88
		57	Qinfeng 2013	42.71	20.69 – 64.72
			Pooled	**247.41**	**178.06 – 316.76**

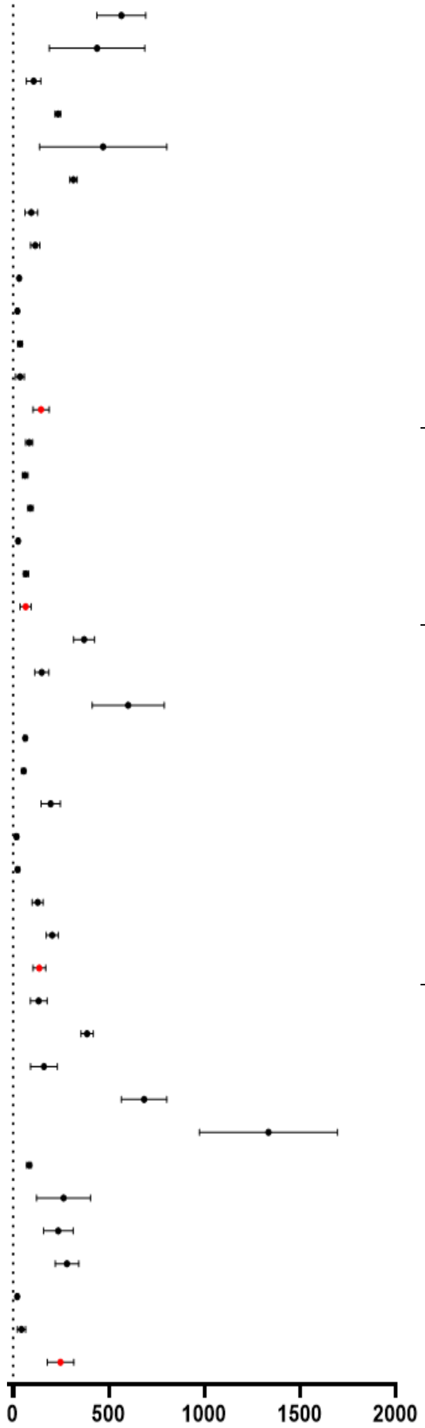

**Stromal**					
Normal	6	5	Rosini 1996	448.70	270.06 – 627.34
		10	Kobayashi 2004	545.00	354.63 – 735.37
		4	Monnier-Benoit 2006	612.33	522.34 – 702.33
		9	Piersma 2007	385.00	322.28 – 447.72
		9	Needergaard 2007	303.00	186.37 – 419.63
		7	Carrero 2015	15.30	7.74 – 22.86
			Pooled	**381.13**	**130.13 – 632.13**
Low grade	5	19	Rosini 1996	257.80	207.75 – 307.85
		9	Monnier-Benoit 2006	529.00	433.74 – 624.26
		5	Monnier-Benoit 2006	707.33	586.27 – 828.39
		26	Bedoya 2013	83.17	61.46 – 104.88
		45	Carrero 2015	51.00	43.55 – 58.45
			Pooled	**303.29**	**193.31 – 413.2**
High grade	9	16	Coleman 1994	1839.00	1532.14 – 2145.86
		19	Rosini 1996	399.40	320.67 – 478.13
		14	Kobayashi 2004	1933.00	1462.24 – 2403.76
		13	Monnier-Benoit 2006	680.33	511.05 – 849.62
		10	Loddenkemper 2009	663.00	453.46 – 872.54
		21	Bedoya 2013	51.97	27.72 – 76.22
		25	Bedoya 2013	80.55	54.74 – 106.36
		10	Carrero 2015	137.70	128.22 – 147.18
		10	Carrero 2015	408.00	344.78 – 471.22
			Pooled	**457.70**	**358.45 – 556.95**
Cancer	11	7	Rosini 1996	487.30	338.18 – 636.42
		48	Bethwaite 1996	2750.00	2604.55 – 2859.45
		11	Monnier-Benoit 2006	859.33	624.99 – 1093.67
		59	Piersma 2007	966.00	803.97 – 1128.03
		102	Needergaard 2007	1378.67	1199.26 – 1558.07
		20	Needergaard 2007	1681.00	1109.64 – 2252.36
		12	Loddenkemper 2009	768.40	531.07 – 1005.73
		10	Shah 2011	217.92	158.89 – 276.96
		30	Shah 2011	219.10	167.30 – 270.89
		24	Bedoya 2013	101.29	58.54 – 144.04
		57	Qinfeng 2013	87.77	63.05 – 112.49
			Pooled	**838.31**	**559.65 – 1116.96**

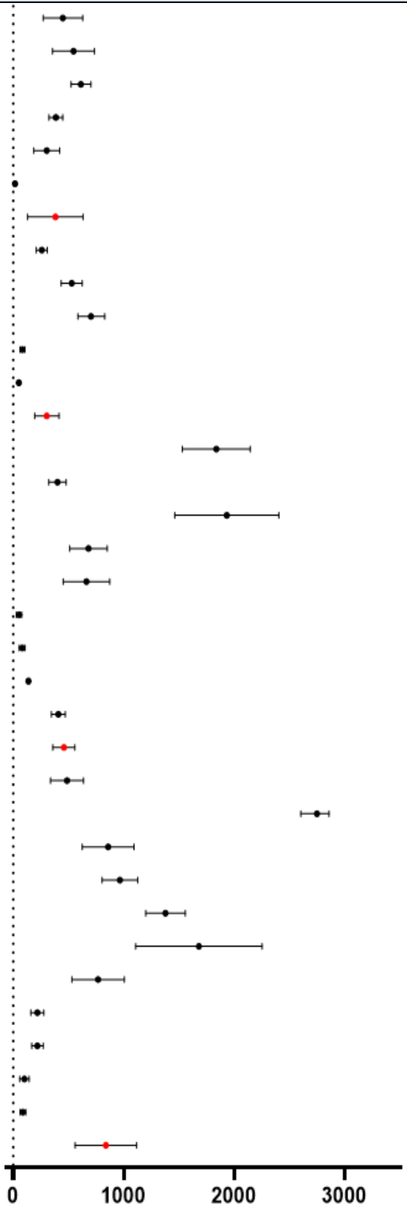

CD4						
**Subgroup**	**Number of studies**	**Sample Size**	**Study authors**	**Random effects model**	**Mean**	**95% CI**
**Total**						
Normal	3	13	Edwards 1995		99.96	64.20 – 135.72
		17	Bell 2000		550.80	453.83 – 647.77
		37	Adurthi 2008		12.00	9.42 - 14.58
			Pooled		**209.39**	**43.80 – 374.98**
Low grade	3	16	Edwards 1995		196.86	156.13 – 237.59
		7	Bontkes 1997		21.86	-5.12 – 48.83
		15	Jaafar 2009		202.30	144.95 – 259.65
			Pooled		**139.00**	**6.52 – 271.47**
High grade	6	20	Edwards 1995		267.24	213.82 – 320.66
		7	Bontkes 1997		29.43	-8.56 – 6.43
		10	Bontkes 1997		25.14	-21.39 – 71.67
		30	Adurthi 2008		206.00	192.76 – 219.24
		15	Jaafar 2009		163.20	130.70 – 195.70
		20	Jaafar 2009		164.90	135.10 – 194.70
			Pooled		**142.80**	**79.25 – 206.36**
Cancer	8	16	Edwards 1995		245.31	192.33 – 298.29
		14	Bontkes 1997		31.86	-37.42 – 101.14
		22	Hachisuga 2001		575.28	451.04 – 699.52
		12	Hachisuga 2001		696.66	440.71 – 952.61
		30	Adurthi 2008		312.00	300.55 – 323.45
		7	Jaafar 2009		333.20	274.43 – 391.97
		15	Jaafar 2009		229.50	183.62 – 275.38
		67	Wang 2014		112.40	96.08 – 128.73
			Pooled		**286.85**	**191.14 – 382.55**

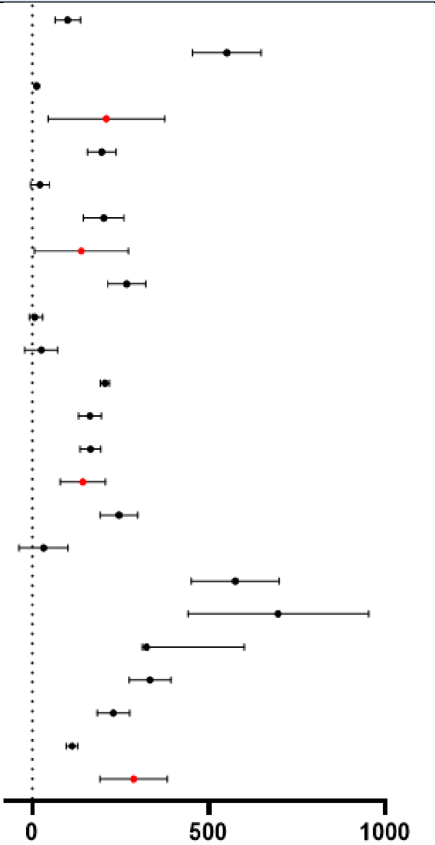

**Epithelial**					
		29	Poppe 1995	281.33	189.94 – 372.73
		9	Poppe 1995	117.67	81.37 – 153.96
		150	Szarewski 2001	107.33	97.97 – 116.70
		11	Kobayashi 2004	62.00	31.86 – 92.14
		16	Pudney 2005	135.00	120.30 – 149.70
Normal	10	4	Monnier-Benoit 2006	44.67	24.34 – 64.99
		9	Nedergaard 2007	20.00	12.74 – 27.26
		115	Jordanova 2008	23.40	21.52 – 25.28
		9	Jaafar 2009	146.20	84.50 – 207.90
		15	Jaafar 2009	110.50	93.29 – 127.71
			Pooled	**93.90**	**63.13 – 124.67**
		9	Monnier-Benoit 2006	37.67	25.08 – 50.25
Low grade	4	5	Monnier-Benoit 2006	25.00	16.56 – 33.44
		115	Woo 2014	15.91	14.80 – 17.02
		26	Bedoya 2013	6.04	4.72 – 7.36
			Pooled	**18.76**	**10.78 – 26.75**
		14	Kobayashi 2004	167.00	82.66 – 251.34
		13	Monnier-Benoit 2006	16.00	11.17 – 20.83
		59	Woo 2014	16.93	16.89 – 16.97
High grade	6	10	Loddenkemper 2009	102.00	57.51 – 146.49
		21	Bedoya 2013	1.20	0.91 – 1.49
		25	Bedoya 2013	5.80	4.45 – 7.15
			Pooled	**16.37**	**5.86 – 26.88**
		11	Monnier-Benoit 2006	14.67	9.85 – 19.48
		102	Nedergaard 2007	110.33	82.01 – 138.65
		20	Nedergaard 2007	207.33	99.23 – 315.44
Cancer	9	115	Jordanova 2008	65.90	53.51 – 78.29
		12	Loddenkemper 2009	147.90	43.17 – 252.63
		10	Shah 2011	83.33	47.90 – 118.77
		30	Shah 2011	101.49	70.48 – 132.50
		24	Bedoya 2013	5.52	4.51 – 6.53
		57	Qinfeng 2013	21.88	18.15 – 25.61
			Pooled	**52.43**	**37.77 – 67.09**

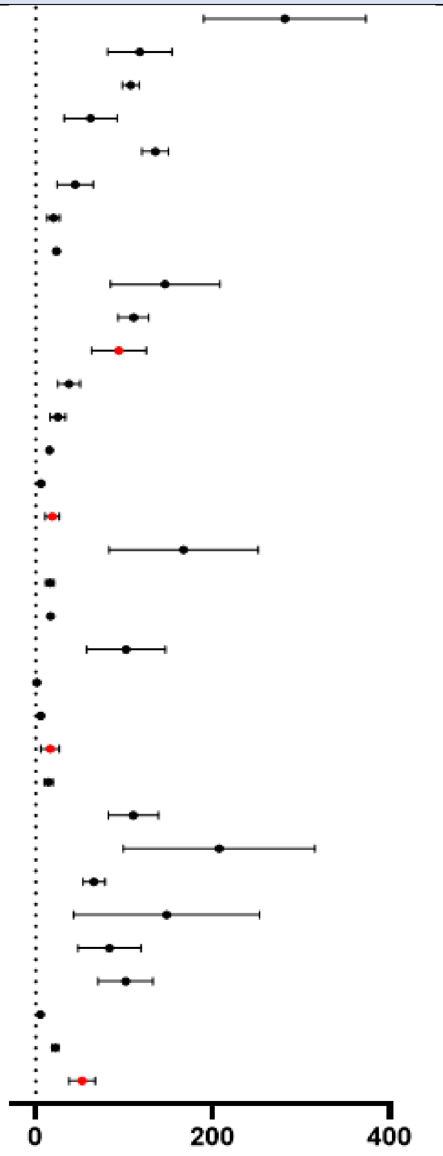

Stromal					
Normal	4	25	Al-Saleh 1998	24.00	18.90 – 29.10
		10	Kobayashi 2004	215.00	117.69 – 312.31
		4	Monnier-Benoit 2006	322.33	247.56 – 397.10
		9	Nedergaard 2007	72.67	39.76 – 105.57
			Pooled	**149.43**	**52.44 – 246.42**
Low grade	5	14	Al-Saleh 1998	27.00	18.62 – 35.38
		9	Monnier-Benoit 2006	360.00	271.92 – 448.08
		5	Monnier-Benoit 2006	360.67	296.39 – 424.94
		115	Woo 2014	85.94	30.73 – 141.14
		26	Bedoya 2013	30.52	23.21 – 37.83
			Pooled	**142.00**	**94.33 – 189.67**
High grade	7	12	Al-Saleh 1998	29.00	20.51 – 37.49
		14	Kobayashi 2004	1083.00	722.09 – 1443.91
		13	Monnier-Benoit 2006	317.67	182.77 – 452.56
		59	Woo 2014	60.08	60.04 – 60.12
		10	Loddenkemper 2009	379.10	180.08 – 578.12
		21	Bedoya 2013	9.45	6.69 – 12.21
		25	Bedoya 2013	20.62	13.92 – 27.32
			Pooled	**59.81**	**27.30 – 92.32**
Cancer	8	11	Monnier-Benoit 2006	306.33	213.97 – 398.70
		102	Nedergaard 2007	265.00	213.68 – 316.32
		20	Nedergaard 2007	722.67	490.23 – 955.11
		12	Loddenkemper 2009	391.00	220.00 – 562.00
		10	Shah 2011	114.95	77.43 – 152.47
		30	Shah 2011	103.63	62.75 – 144.51
		24	Bedoya 2013	29.71	19.89 – 39.53
		57	Qinfeng 2013	48.96	28.32 – 69.60
			Pooled	**185.06**	**121.50 – 248.61**

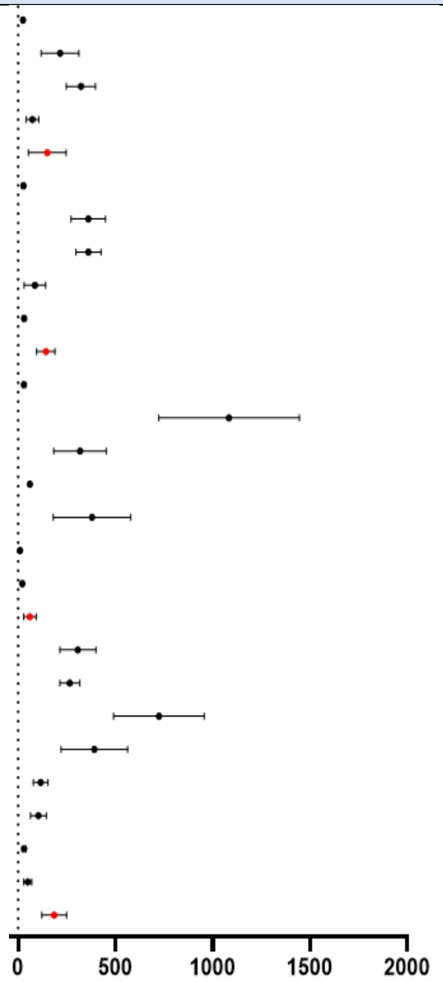

CD8

Subgroup	Number of studies	Sample Size	Study authors	Random effects model	Mean	95% CI
**Total**						
Normal	3	13	Edwards 1995		57.12	43.26 – 70.98
		16	Bell 2000		311.10	286.37 – 335.83
		37	Adurthi 2008		16.00	12.78 – 19.22
			Pooled		**127.33**	**12.78 – 247.91**
Low grade	3	16	Edwards 1995		153.00	119.26 – 186.74
		7	Bontkes 1997		56.03	36.36 – 75.70
		15	Jaafar 2009		215.90	177.66 – 254.14
			Pooled		**140.61**	**42.93 – 238.29**
High grade	6	20	Edwards 1995		207.06	173.53 – 240.59
		7	Bontkes 1997		117.74	88.32 – 147.15
		10	Bontkes 1997		75.94	45.59 – 106.29
		30	Adurthi 2008		235.00	223.91 – 246.09
		15	Jaafar 2009		205.70	173.20 – 238.20
		20	Jaafar 2009		192.10	163.95 – 220.25
			Pooled		**172.66**	**120.31 – 225.01**
Cancer	8	16	Edwards 1995		538.05	463.33 – 612.77
		14	Bontkes 1997		131.26	81.25 – 181.28
		22	Hachisuga 2001		778.77	548.61 – 1008.93
		12	Hachisuga 2001		584.97	337.68 – 832.26
		30	Adurthi 2008		387.00	379.49 – 394.51
		7	Jaafar 2009		450.50	159.45 – 741.55
		15	Jaafar 2009		399.50	248.47 – 550.53
		67	Wang 2014		1231.75	1077.28 – 1386.22
			Pooled		**551.80**	**393.94 – 709.66**

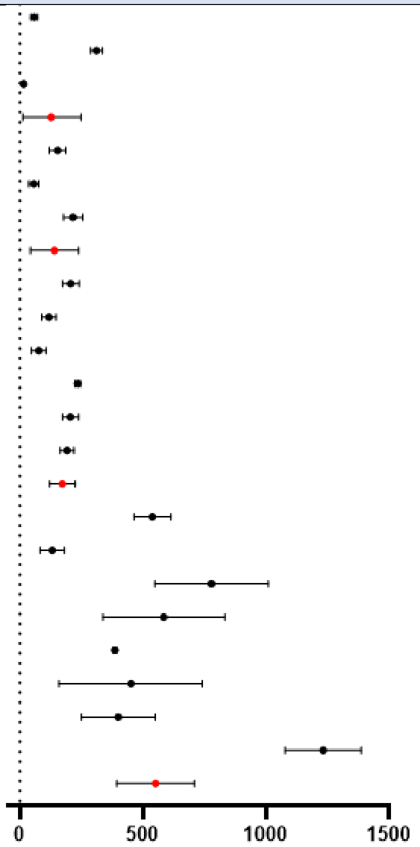

Epithelial					
Normal	11	29	Poppe 1995	283.67	194.43 - 372.90
		9	Poppe 1995	321.00	73.71 – 568.29
		150	Szarewski 2001	126.33	115.78 – 136.88
		11	Kobayashi 2004	408.00	77.07 – 738.93
		15	Pudney 2005	180.00	167.75 – 192.25
		4	Monnier-Benoit 2006	50.00	23.87 – 76.13
		9	Piersma 2007	75.00	42.33 – 107.67
		9	Nedergaard 2007	94.00	64.00 – 124.00
		115	Jordanova 2008	7.31	6.15 – 8.47
		9	Jaafar 2009	187.00	145.04 – 228.96
		15	Jaafar 2009	125.80	89.48 – 162.12
			Pooled	**137.48**	**75.87 – 199.09**
Low grade	4	9	Monnier-Benoit 2006	24.33	17.07 – 31.59
		5	Monnier-Benoit 2006	64.67	54.28 – 75.06
		115	Woo 2008	41.72	32.91 – 50.53
		26	Bedoya 2013	19.71	14.42 – 25.00
			Pooled	**37.21**	**19.33 – 55.10**
High grade	6	13	Kobayashi 2004	434.00	259.51 – 608.49
		13	Monnier-Benoit 2006	47.33	41.70 – 52.97
		59	Woo 2008	37.59	25.83 – 49.34
		10	Loddenkemper 2009	93.50	70.09 – 116.91
		21	Bedoya 2013	15.95	7.58 – 24.32
		25	Bedoya 2013	17.70	8.98 – 26.42
			Pooled	**45.55**	**25.07 – 66.04**
Cancer	12	11	Monnier-Benoit 2006	146.33	76.29 – 216.37
		59	Piersma 2007	135.00	95.45 – 174.55
		102	Nedergaard 2007	496.00	401.99 – 590.01
		20	Nedergaard 2007	620.33	403.48 – 837.19
		115	Jordanova 2008	17.34	11.98 – 22.70
		12	Loddenkemper 2009	115.60	21.55 – 209.65
		10	Shah 2011	152.59	84.00 – 221.18
		30	Shah 2011	179.62	126.68 – 232.56
		24	Bedoya 2013	15.42	10.58 – 20.26
		57	Qinfeng 2013	20.83	4.92 – 36.74
		31	Heeren 2018	249.50	153.40 – 345.60
		137	Liang 2018	108.77	90.79 – 126.76
			Pooled	**125.96**	**97.32 – 154.60**

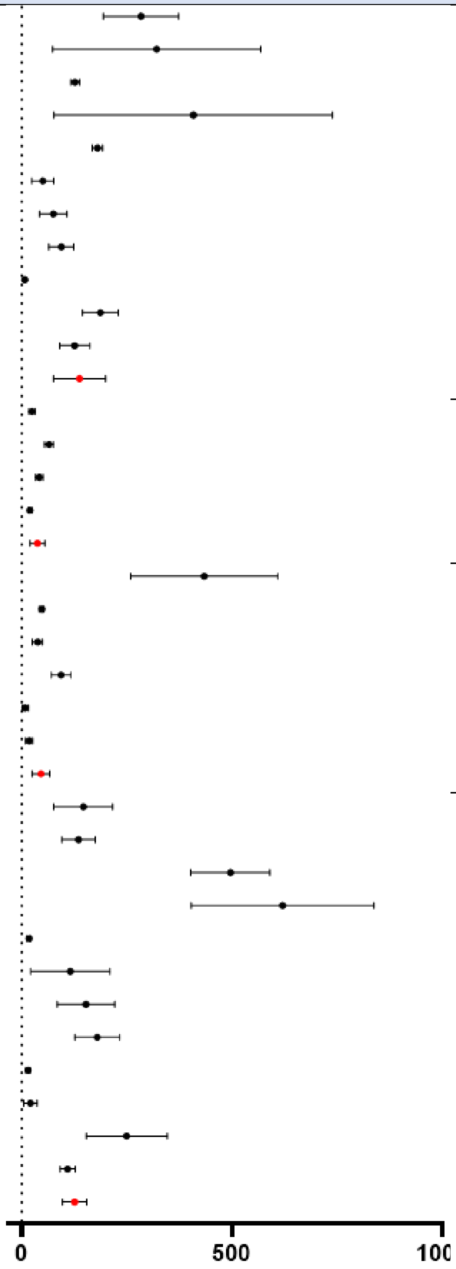

**Stromal**					
Normal	3	10	Kobayashi 2004	330.00	166.37 – 493.63
		4	Monnier-Benoit 2006	290.00	239.91 – 340.09
		9	Nedergaard 2007	151.67	85.85 – 217.48
			Pooled	**246.80**	**135.62 – 357.99**
Low grade	4	9	Monnier-Benoit 2006	169.00	132.70 – 205.30
		5	Monnier-Benoit 2006	346.67	244.08 – 449.25
		115	Woo 2008	64.06	-96.55 – 224.66
		26	Bedoya 2013	52.65	32.21 – 73.09
			Pooled	**157.48**	**50.48 – 264.48**
High grade	6	13	Kobayashi 2004	850.00	536.34 – 1163.66
		13	Monnier-Benoit 2006	362.67	260.39 – 464.94
		59	Woo 2008	80.63	64.24 – 97.02
		10	Loddenkemper 2009	283.90	218.34 – 349.46
		21	Bedoya 2013	42.52	18.42 – 66.62
		25	Bedoya 2013	59.93	35.00 – 84.86
			Pooled	**173.93**	**107.50 – 240.43**
Cancer	10	11	Monnier-Benoit 2006	553.00	337.63 – 768.37
		102	Nedergaard 2007	796.33	683.34 – 909.32
		20	Nedergaard 2007	1279.33	899.18 – 1659.48
		12	Loddenkemper 2009	377.40	212.82 – 541.98
		10	Shah 2011	102.97	57.39 – 148.55
		30	Shah 2011	115.46	83.65 – 147.27
		24	Bedoya 2013	71.58	29.97 – 113.19
		57	Qinfeng 2013	38.81	6.94 – 70.68
		31	Heeren 2018	863.60	686.75 – 1040.25
		137	Liang 2018	375.85	310.73 – 440.97
			Pooled	**395.33**	**273.75 – 516.90**

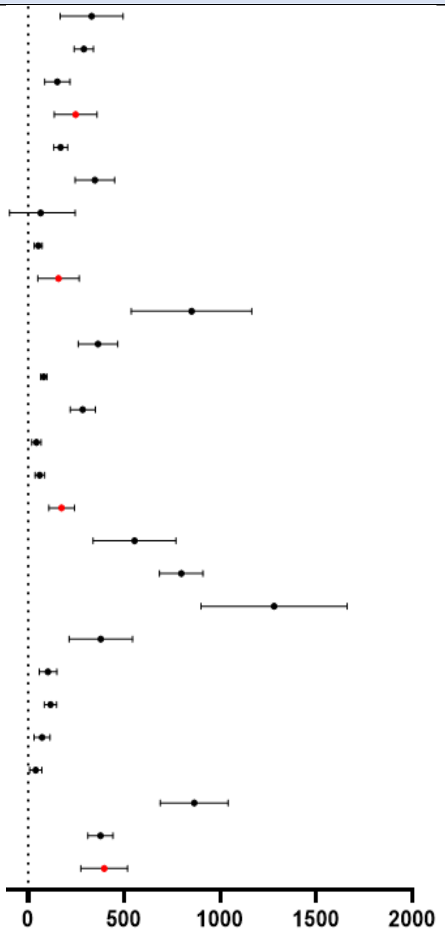

CD4:CD8 Ratio						
**Subgroup**	**Number of studies**	**Sample Size**	**Study authors**	**Random effects model**	**Mean**	**95% CI**
**Total**						
Normal	4	13	Edwards 1995		1.75	0.99 – 2.51
		11	Bell 2000		1.11	0.89 – 1.33
		37	Adurthi 2008		0.75	0.53 – 0.97
		6	Kuppers 1998		0.62	0.47 – 0.77
			Pooled		**0.93**	**0.61 – 1.24**
Low grade	4	16	Edwards 1995		1.29	0.90 – 1.68
		7	Bontkes 1997		0.39	-0.11 – 0.89
		15	Jaafar 2009		0.94	0.62 – 1.25
		5	Kuppers 1998		0.42	0.31 – 0.53
			Pooled		**0.75**	**0.33 – 1.18**
High grade	8	20	Edwards 1995		1.29	0.96 – 1.62
		7	Bontkes 1997		0.25	-0.08 – 0.58
		10	Bontkes 1997		0.33	-0.30 – 0.96
		30	Adurthi 2008		0.88	0.81 – 0.95
		15	Jaafar 2009		0.79	0.59 – 1.00
		20	Jaafar 2009		0.86	0.66 – 1.06
		4	Kuppers 1998		0.51	0.37 – 0.65
		13	Kuppers 1998		0.50	0.37 – 0.63
			Pooled		**0.70**	**0.51 – 0.88**
Cancer	9	16	Edwards 1995		0.46	0.34 – 0.57
		14	Bontkes 1997		0.24	-0.29 – 0.78
		22	Hachisuga 2001		0.74	0.47 – 1.01
		12	Hachisuga 2001		1.19	0.52 – 1.86
		30	Adurthi 2008		0.81	0.77 – 0.84
		7	Jaafar 2009		0.74	0.24 – 1.23
		15	Jaafar 2009		0.57	0.33 – 0.82
		67	Wang 2014		10.96	8.86 – 13.06
		9	Kuppers 1998		0.11	0.02 – 0.20
			Pooled		**0.80**	**0.47 – 1.13**

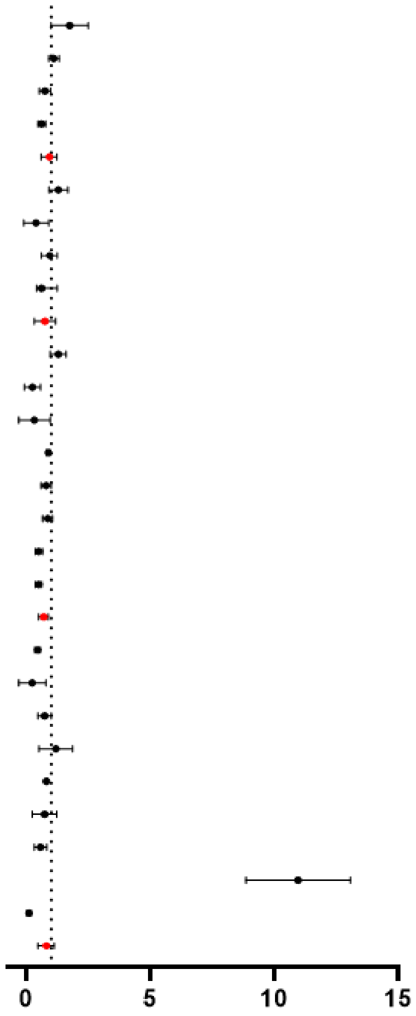

Epithelial

		29	Poppe 1995	0.99	0.54 – 1.44
		9	Poppe 1995	0.37	0.06 – 0.67
		150	Szarewski 2001	0.85	0.75 – 0.95
		11	Kobayashi 2004	0.15	0.01 – 0.30
		16	Pudney 2005	0.75	0.65 – 0.85
Normal	10	4	Monnier-Benoit 2006	0.41	0.28 – 0.54
		9	Nedergaard 2007	0.21	0.11 – 0.32
		115	Jordanova 2008	3.20	2.76 – 3.64
		9	Jaafar 2009	0.78	0.41 -1.16
		15	Jaafar 2009	0.88	0.59 – 1.17
			Pooled	**0.81**	**0.53 – 1.10**
		9	Monnier-Benoit 2006	0.60	0.43 – 0.77
Low grade	4	5	Monnier-Benoit 2006	0.78	0.68 – 0.87
		115	Woo 2008	4.30	3.53 – 5.07
		26	Bedoya 2013	0.35	0.28 – 0.42
			Pooled	**1.17**	**0.71 – 1.63**
		13	Kobayashi 2004	0.38	0.13 – 0.64
		13	Monnier-Benoit 2006	0.76	0.61 – 0.91
		59	Woo 2008	3.62	1.95 – 5.29
High grade	6	10	Loddenkemper 2009	1.09	0.54 – 1.64
		21	Bedoya 2013	0.19	0.14 – 0.24
		25	Bedoya 2013	0.83	0.73 – 0.93
			Pooled	**0.75**	**0.37 – 1.12**
		11	Monnier-Benoit 2006	0.27	-0.01 – 0.56
		102	Nedergaard 2007	0.22	0.15 – 0.29
		20	Nedergaard 2007	0.33	0.12 – 0.54
Cancer	9	115	Jordanova 2008	3.80	2.87 – 4.73
		12	Loddenkemper 2009	1.28	-0.10 – 2.66
		10	Shah 2011	0.70	0.43 – 0.97
		30	Shah 2011	0.94	0.55 – 1.33
		24	Bedoya 2013	0.42	0.33 – 0.51
		57	Qinfeng 2013	1.05	-0.03 – 2.13
			Pooled	**0.66**	**0.42 – 0.91**

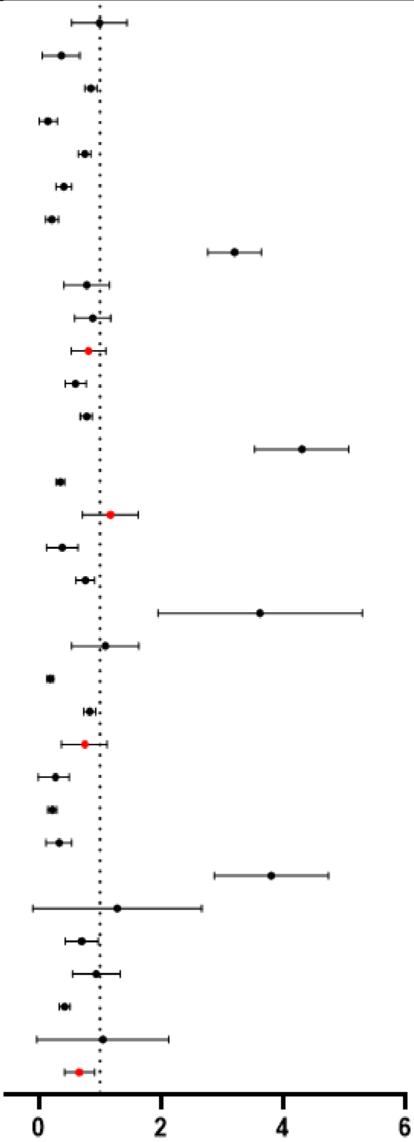

**Stromal**					
Normal	3	10	Kobayashi 2004	0.65	0.21 – 1.09
		4	Monnier-Benoit 2006	0.75	0.66 – 0.85
		9	Nedergaard 2007	0.48	0.18 – 0.78
			Pooled	**0.68**	**0.51 – 0.85**
Low grade	3	9	Monnier-Benoit 2006	1.27	1.14 – 1.41
		5	Monnier-Benoit 2006	0.96	0.87 – 1.05
		26	Bedoya 2013	0.53	0.41 – 0.65
			Pooled	**0.92**	**0.54 – 1.30**
High grade	5	13	Kobayashi 2004	1.27	0.63 – 1.92
		13	Monnier-Benoit 2006	0.91	0.85 – 0.97
		10	Loddenkemper 2009	1.34	0.57 – 2.10
		21	Bedoya 2013	0.62	0.50 – 0.74
		25	Bedoya 2013	3.59	3.12 – 4.06
			Pooled	**1.50**	**0.98 – 2.03**
Cancer	8	11	Monnier-Benoit 2006	0.37	0.23 – 0.52
		102	Nedergaard 2007	0.33	0.25 – 0.41
		20	Nedergaard 2007	0.56	0.32 – 0.81
		12	Loddenkemper 2009	1.04	0.40 – 1.68
		10	Shah 2011	1.68	0.88 – 2.48
		30	Shah 2011	1.25	0.82 – 1.68
		24	Bedoya 2013	1.50	1.15 – 1.85
		57	Qinfeng 2013	1.26	0.53 – 1.99
			Pooled	**0.90**	**0.60 – 1.20**

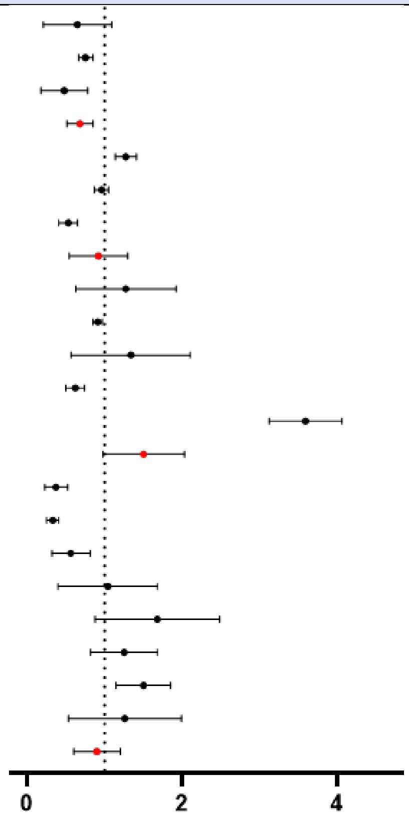

Foxp3						
**Subgroup**	**Number of studies**	**Sample Size**	**Study authors**	**Random effects model**	**Mean**	**95% CI**
**Total**						
Normal	2	18	Hou 2012	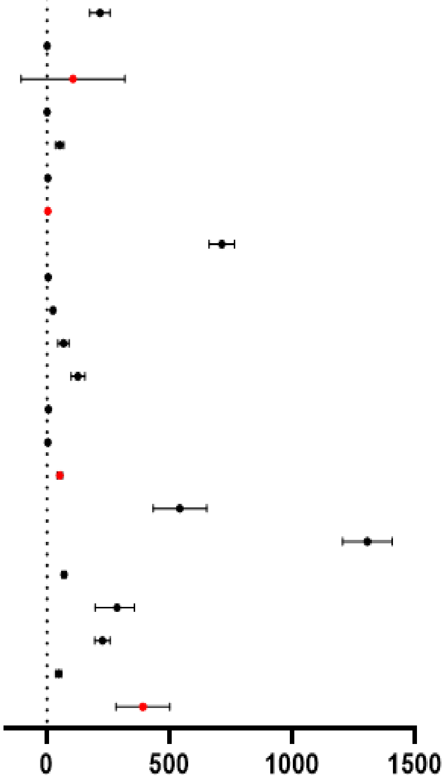	215.73	173.98 - 257.48
		8	Adurthi 2008		0.12	-0.12 - 0.37
			Pooled		**106.88**	**-104.40 - 318.15**
Low grade	3	21	Prata 2015		1.00	0.79 – 1.21
		1	Jaafar 2009		52.70	35.49 – 69.91
		11	Adurthi 2008		3.40	2.69 – 4.11
			Pooled		**3.75**	**0.62 – 6.88**
High grade	7	28	Hou 2012		713.03	660.80 – 765.26
		20	Prata 2015		5.00	4.12 – 5.88
		16	Prata 2015		25.00	20.10 – 29.90
		15	Jaafar 2009		68.00	45.06 – 90.94
		20	Jaafar 2009		127.50	99.35 – 155.65
		30	Adurthi 2008		6.00	4.57 – 7.43
		10	Adurthi 2008		4.30	2.81 – 5.79
			Pooled		**52.32**	**41.17 – 63.48**
Cancer	6	67	Wang 2014		541.77	432.90 – 650.64
		46	Hou 2012		1305.70	1204.81- 1406.60
		22	Prata 2015		70.00	61.64 – 78.36
		7	Jaafar 2009		285.60	196.05 – 375.15
		15	Jaafar 2009		226.10	195.51 – 256.69
		30	Adurthi 2008		48.00	36.19 – 59.81
			Pooled		**391.32**	**282.19 – 500.45**
**Epithelial**						
Normal	3	9	Jaafar 2009	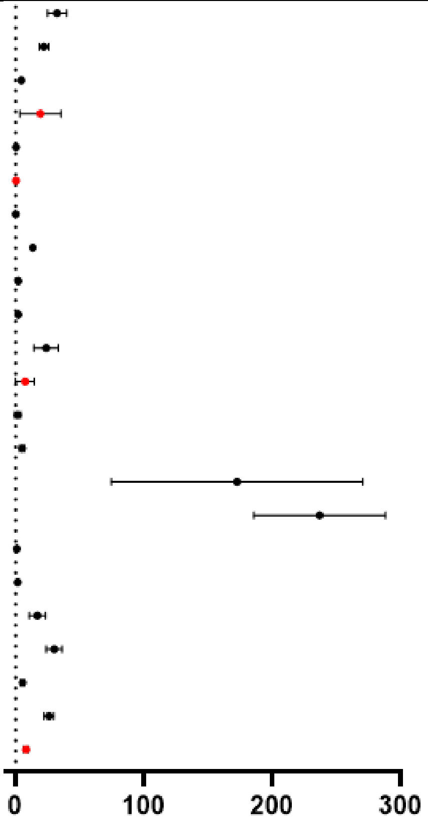	32.30	24.90 – 39.70
		15	Jaafar 2009		22.10	18.28 - 25.92
		115	Jordanova 2008		4.59	3.84 – 5.34
			Pooled		**19.36**	**3.22 – 35.50**
Low grade	1	26	Bedoya 2013		0.38	0.33 - 0.43
			Pooled		**0.38**	**0.33 - 0.43**
High grade	5	31	Nakamura 2007		0.0	-0.13 – 0.13
		59	Woo 2008		13.46	13.36 – 13.57
		21	Bedoya 2013		2.02	1.65 – 2.39
		25	Bedoya 2013		2.06	1.84 – 2.28
		10	Loddenkemper 2009		23.80	14.43 – 33.17
			Pooled		**7.44**	**0.08 – 14.79**
Cancer	10	10	Nakamura 2007		1.68	-0.80 – 4.16
		18	Nakamura 2007		5.15	3.01 – 7.29
		10	Shah 2011		172.53	74.73 – 270.33
		30	Shah 2011		236.84	185.62 – 288.07
		59	Piersma 2007		1.00	0.87 – 1.13
		24	Bedoya 2013		1.71	1.42 – 2.00
		12	Loddenkemper 2009		17.00	10.59 – 23.41
		115	Jordanova 2008		30.09	23.94 – 36.24
		57	Qinfeng 2013		5.38	3.57 – 7.18
		137	Liang 2018		26.16	22.44 – 29.88
			Pooled		**8.12**	**6.25 – 9.99**

**Stromal**					
Normal	0	-	-	-	-
Low grade	1	26	Bedoya 2013	6.76	5.61 – 7.91
			Pooled	**6.76**	**5.61 – 7.91**
High grade	5	31	Nakamura 2007	11.68	9.49 – 13.86
		59	Nakamura 2007	48.65	-1.63 – 98.93
		21	Bedoya 2013	3.06	1.92 – 4.20
		25	Bedoya 2013	10.04	8.87 – 11.21
		10	Loddenkemper 2009	166.60	33.14 – 300.06
			Pooled	**8.96**	**3.42 – 14.51**
Cancer	9	10	Nakamura 2007	53.75	45.53 – 61.98
		18	Nakamura 2007	43.55	38.69 – 48.42
		10	Shah 2011	117.56	60.09 – 175.02
		30	Shah 2011	179.32	168.09 – 190.54
		59	Piersma 2007	12.00	10.72 – 13.28
		24	Bedoya 2013	5.48	4.58 – 6.38
		12	Loddenkemper 2009	117.30	38.21 – 196.39
		57	Qinfeng 2013	4.23	3.07 – 5.39
		137	Liang 2018	111.12	88.79 – 133.44
			Pooled	**56.03**	**44.97 – 67.09**

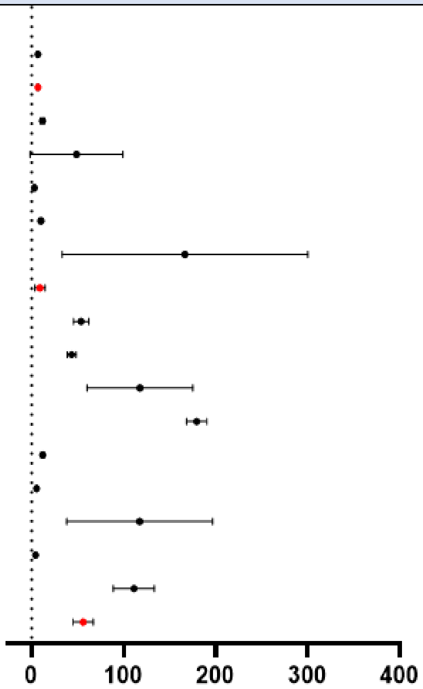

Figure S1. Full Forest Plots. Forest plots of each population subset included in the quantitative meta-analysis of infiltrating CD3, CD4, CD8, the CD4:CD8 ratio, and FoxP3 in normal cervix, low grade cervical intraepithelial neoplasia (CIN), high grade CIN, and cervical cancer tissue. Abbreviations: CI, confidence interval.

Figure S2. Tests of Variance

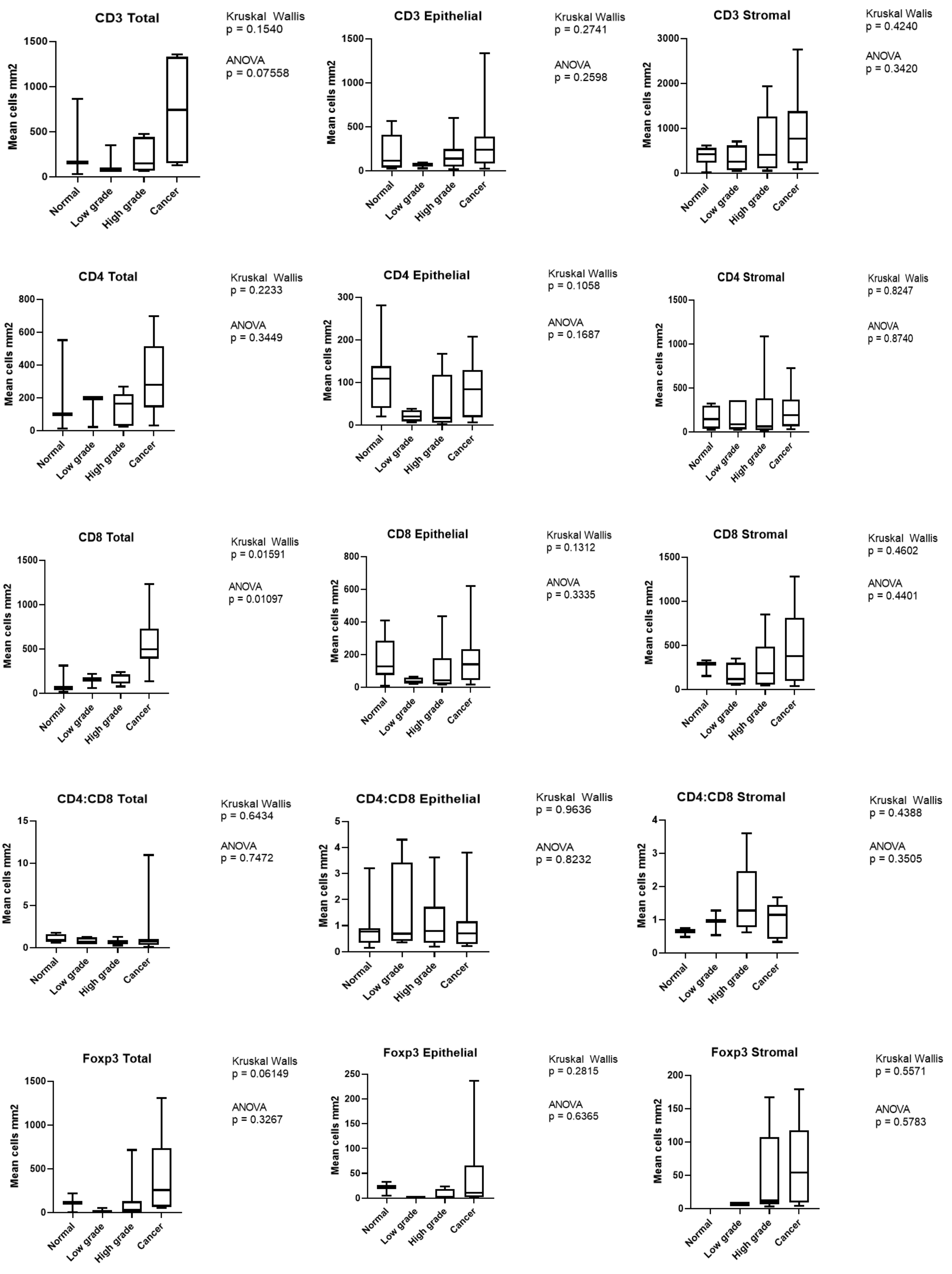

Figure S2. Tests of Variance. Pairwise nonparametric (Kruskal Wallis) and parametric (ANOVA) tests of variance showed comparable results for each T-cell subset and ratio. Only CD8 total was nominally significant ($p<0.05$) for both tests. Pairwise nonparametric Mann-Whitney tests reveal that this result was driven by significant differences between cancer and each other disease stage.

Table S1. Studies Included in Quantitative Meta-Analysis

			Disease level					Tissue type			Markers of interest							Age		Method
First Author	Year	PMID	Normal	LGCIN	HGCIN	Cancer	Other	Epithelial	Stromal	Total	CD4	CD8	CD3	CD4:CD8	CD56	Foxp3	CD25	Years	Reporting metric	IHC/IF
Abdulhaqq	2016	26555708	13					X			X							21	Minimum	IF
Adurthi	2008	18593438	37		30	30	37			X	X	X	I	I		X	X	26-76	Range	IHC
Ahmed	2001	11439171	10					X	X		I	X	I	X				28-33	Range	IF
al-Saleh	1998	9614381	34	14	12				X		X							NR	NR	IHC
Ancuta	2009	19942961				61		UNK	UNK	UNK			X					36.4	Mean	IHC
Bedoya	2013	22290207		26	46	24		X	X		X	X	I	X		X	X	33.7/33.6/47/47.2	Mean (CIN1/CIN2/CIN3/cancer)	IHC
Bell	2000	10684703	17				6			X	X	X	I	X				39.3/27.3/26.1	Mean (Normal/CIN HIV-/CIN HIV+)	IHC
Bethwaite	1996	9007950				64		X	X				X					43.7	Mean	IHC
Bontkes	1997	9374383		7	17	14				X	I	X	X	I				NR	NR	IHC
Brustmann	2015	25675190	54	25	44	64		X	X			X						NR	NR	IHC
Carrero	2015	25661067	7	45	10			X	X				X					NR	NR	IF
Chen	2006	16681759				55		X	X			X						NR	NR	IHC
Coleman	1994	8314316			16			X	X				X					NR	NR	IHC
Dietl	1991	1671375				10		X	X		X	X	X					48	Median	IHC
Edwards	1995	8620416	13	16	20	16				X	X	X	I	I				NR	NR; 15 years older in cancer than CIN	IHC
Enwere	2017	28059093				111				X		X						44	Median	IHC
Ferguson	1985	2415145	13			10		X			X	X	X					31-77	Range	IHC
Ferrandina	2006	16609015				27			X		X	X	X				X	51/58	Median (treated/untreated)	IHC
Gey	2003	12628838				12				X			X					NR	NR	IHC
Hachisuga	2001	11549855				34				X	X	X	I	I				53	Mean	IHC
Heeren	2018	30050535				35		X	X			X						4.9	NR	IHC and IF
Hilders	1993	8264228				30		X	X		X	X	X		X			NR	NR	IHC
Hirbod	2013	24006463	20					X			X							42/38/42	Median (HIV+ FSW/HIV-/HIV- FSW)	IHC
Hou	2012	22820395	18		28	46				X						X		45/39/46	Median (cancer/CIN3/normal)	IHC
Hu	2015	25885042			13					X			X					38.2/36.9	Median (HPV+/HPV-)	IHC
Jaafar	2009	19808652	9	15	35	22	6	X		X	X	X	X	I		X		NR	NR	IF
Jordanova	2008	18381941	115			115		X			X	I	I	X				48.5/46	Mean (patients)/median (controls)	IF (CD8); IHC (FoxP3)
Kobayashi	2004	15374995	21		14			X	X		X	X	I	I	X			51/33/32	Mean (HIV- normal/HIV CIN/HIV+ CIN)	IHC
Kuppers	1998	25951354	6	5	17	9				X	X	X		X				NR	NR	IHC
Li	2014	25423704	24	28	50	24				X	X							NR	NR	IHC
Liang	2018	30474571				137		X	X			X				X		NR	NR	IHC
Loddenkemper	2009	19514119			10	12		X	X		X	X	I	I		X		NR	NR	IHC
Lucena	2016	26545568		6	31			UNK	UNK	UNK	X	X	X		X			32.8/35.3	Mean	IHC
Maldonado	2014	24477000	12					X	X			X				X		29	Mean	IHC
Maluf	2008	18343936			35				X			X	X					34.9	Mean	IHC
Monnier-Benoit	2006	16427684	4	14	13	11		X	X		X	X	I	X				44/35/44	Median (normal/CIN/cancer)	IHC
Munk	2012	23017821					162		X		X							25-40	Range	IHC
Nakamura	2007	17433037	24		31	28	13	X	X							X	X	NR	NR	IHC/IF
Nedergaard	2007	17940503				102		X	X		X	X	X	I				NR	NR	IHC
Nedergaard	2007	18184401	9			20		X	x		X	X	X	I				31.5	Median	IHC
Olaitan	1996	8805867	5							X				X				37	Mean	IHC
Origoni	2013	24455729			34			X	X		X	X						NR	NR	IHC
Ovestad	2011	21421698			55			X	X			X					X	35.2/48.6	Mean (CIN-cancer/normal)	IHC
Peghini	2012	22749886		21	34	8					X						X	44/46	Median (cancer/normal)	IF/IHC
Piersma	2007	17210718	9			59		X	X			X	X			X		36/43	Mean (nonsmokers/smokers)	IHC
Poppe	1995	7890250	38					X			X	X	I	I				49/3/41/45/45	Mean (normal/CIN1/CIN2/CIN3/cancer)	IHC
Prata	2015	26059395	5	21	36	22				X						X		43	Mean	IHC
Pudney	2005	16093359	16					X			X	X	I	I				40	Median	IF
Punt	2015	25795131				67		X	X				X			X		51	Median	IHC
Qinfeng	2013	23510275				57		X	X		X	X	I	I				44	Mean	IHC
Roncalli	1988	2448545					18	X	X		X	X	I	I				31.2/32.3/33.4	Mean (HIV-/HIV+ high CD4/HIV+ low CD4)	IHC
Rosini	1996	8760019	5	19	19	7		X	X				X					47	Mean	IHC
Shah	2011	21200385				40		X	X		X	X	I	X		X		47	Median	IHC
Silva	2010	20613932	20		19	19		X	X			X	x					43.9/35.5/50	Mean (normal/CIN3/cancer)	IHC
Srivani	2003	12801265	3	6	13	32	2			X	X	X	X					42.3-55.4	Range of mean ages listed for 8 disease stages	IHC
Szarewski	2001	11281472	150					X			X	X	I	I				35	Mean	IHC
Varynen	1985	2989155	166	62	32	3				X	X	X	X	X				25-29	Median	IHC
Viac	1990	2168858	5		18			X	X		X	X						20-60	Range (hgCIN)	IF
Wang	2014	25446402				67				X	X	X	I	I		X		43	Mean	IF
White	1997	9138451	29							X			X					NR	NR	IF
Woo	2008	19035938		59	115			X	X		X	X	I	X	X	X		20-30	Range	IHC

Table S1. Studies Included in Quantitative Meta-Analysis. Studies included in the quantitative meta-analyses are listed, including record identification information. The numbers of patient samples at each disease stage and which markers and tissues types were included are also indicated. One sample per patient was included from studies that took multiple samples. Directly reported measurements are indicated with an “X,” imputed measurements are indicated with an “I,” and studies with unknown tissue type are indicated with “UNK.” In the meta-analysis unknown tissue type was assumed to be total (see methods). Abbreviations: PMID, PubMed identification number; LG, low grade; CIN, cervical intraepithelial neoplasia; HG, high grade; UNK, unknown; I, imputed; NR, not reported; IHC, immunohistochemistry; IF, immunofluorescence

Table S2. Quality Review

First author	Year	PMID	Study design	Risk of confounding	Confounding notes	Risk of selection bias	Selection bias notes	Risk of information bias	Information bias notes	Follow-up: Years	Follow-up: Reporting metric	Included in datasets: Meta-analysis	Included in datasets: Longitudinal analysis	Included in datasets: CD25 analysis
Abdulhaqq	2016	26555708	Cross-sectional	L	No risk of confounding by HIV status (since all women were determined to be HIV free); low risk of residual confounding	L	Selection into the study described well, special population	L	Standard procedure for all samples, unlikely to result in bias by investigators			Yes		
Adurthi	2008	18593438	Cross-sectional	U	HIV status not reported	L	Selection into the study described well, no concern of bias	L	Manual cell counting, but unlikely related to exposure/outcome overall (any misclassification considered random)			Yes		Yes
Ahmed	2001	11439171	Cross-sectional	U	"all stages of [HIV] disease" - women with advanced HIV may have a different immune profile than women recently infected. 2 women were on antiretroviral treatment treatment	L	Selection into the study described well, special population	L	Standard procedure for all samples, unlikely to result in bias by investigators			Yes		
al-Saleh	1998	9614381	Cross-sectional	U	Study had info on specific HPVs but did not stratify for CD3 analysis; no info on HIV	U	Selection not discussed	L	Method well-described, used control samples,likely low bias			Yes		
Ancuta	2009	19942961	Cross-sectional	L	No HIV information, but prevalence of HIV presumed to be low in population and unlikely to cause bias	L	Risk of selection bias low as all had cancer and selection not expected to be related to outcome	U	Very little information on IHC methods, and no discussion of blinding or automated analysis			Yes		
Ancuta	2014	25329108	Cohort	U	No discussion of potential confounders such as age, HIV status	U	Did not state how 18 cases were selected out of original cohort of 61	U	No information given, possible bias by IHC reviewers since analysis was retrospective	No data			Yes	
Bedoya	2013	22290207	Cross-sectional	L	No HIV information, but prevalence of HIV presumed to be low in population and unlikely to cause bias	L	Selection not discussed, but the high number of samples can negate the risk of selection bias	L	Two separate pathologists independently evaluated samples, staining procedure was standard			Yes		Yes
Bell	2000	10684703	Cross-sectional	U	HIV accounted for in this study, but stage of HIV disease not discussed	H	Low sample count, sampling not discussed in detail. It is very possible that selection into this study was not random and that the low number of samples contributed to a lot of variation in outcome	L	Automated method used			Yes		
Bethwaite	1996	9007950	Cohort baseline	L	Study reported mean CD3 in multiple subgroups (tumor /grade/age at DX/LCSI/lymph nodes/local or nonlocal disease). Risk of HIV low and unlikely to affect results	L	It is unlikely that there was a selection bias when compiling the samples, as they were complied for a separate study with no knowledge that eventual immune infiltrate information would be retrieved.	L	Automated CD3 counts, same method across all samples	5.2	Mean	Yes	Yes	
Bontkes	1997	9374383	Cross-sectional	L	No HIV information, but prevalence of HIV presumed to be low in population and unlikely to cause bias	L	Selction procedure specifically notes random selection	L	Three independent researchers evaluated results			Yes		
Brustmann	2015	25675190	Cross-sectional	L	No HIV information, but prevalence of HIV presumed to be low in population and unlikely to cause bias	L	Selection procedure not well-defined, but high number of samples may lower risk of selection bias	L	Investigator blinded to all clinical data			Yes		
Carrero	2015	25661067	Cross-sectional	L	HIV accounted for in this study, all were tested and negative; additional factors like immunosuppresants and pregnancy were also accounted for	L	The selection of patients is well-described, but whether they were randomly selected is unknown. For this study, I would not be concerned about the outcome per category, but statistical comparisons with the low number of controls	U	Very little information on IF methods			Yes		
Chen	2006	16681759	Cross-sectional	L	Study reviewed expression in many subgroups; risk of bias is low	L	All had adenocarcinomas and likelihod of selection bias is low	L	Independent observers without knowledge of clinical information reviewed the samples			Yes		
Coleman	1994	8314316	Cross-sectional	L	Patients had no infection (other than HPV) or inlammation; HIV not assessed but low prevalence in the population lowers risk that it affected any results	U	Selection not well-described	H	"arbitrary grading scale"			Yes		
Dietl	1991	1671375	Cross-sectional	L	HIV not assessed but likely did not affect results	L	Low sample count, but all had adenocarcinoma. Since disease stage all the same, sampling bias unlikely	U	Semiquantitative and subjective system to rate infiltration			Yes		
Edwards	1995	8620416	Cohort	H	Posisble confounding by age, HIV status, HPV status (for normal category)	H	Insufficient details provided on criteria for tissue selection; patients without known recurrence were not followed up	L	Cell counter blinded to diagnosis	2	NR	Yes	Yes	
Enwere	2017	28059093	Cohort	U	No data on potential confounders including HIV status, age	U	No details provided on selection criteria	U	Does not state whether pathologist counting CD8 cells was blinded to outcome	5	Maximum	Yes	Yes	
Ferguson	1985	2415145	Cross-sectional	U	Possible confounding due to indication for hysterectomy (for normal tissue)	U	Insufficient details provided to evaluate	U	Does not state whether cell scorer blinded to ourocme			Yes		
Ferrandina	2006	16609015	Clinical trial baseline	L	Excluded based on likely confounders	L		L	Pathologist analyses blinded appropriately			Yes		Yes
Gey	2003	12628838	Cross-sectional	U	Possible confounding by HIV status	U	Insufficient details provided to evaluate	U	Fields likely not chosen truly randomly, not stated whether cell counting blinded appropriately			Yes		
Goncalves	2009	19689792	Cross-sectional	L	Stratified by HIV status, excluded for other likely confounders	L	No likely sources of selection bias, patients recruited sequentially	U	Subjective field selection and counting criteria raise questions, no indication whether reviewers were blinded					Yes
Grochot	2019	31191020	Cohort	U	Possible confounding by HIV status	L	All cases in range included, excluded cases not likely to due to bias	U	Not stated whether pathologists blinded to outcome	2.2	Median		Yes	

Hachisuga	2001	11549855	Cohort baseline	U	Possible confounding by HIV status	U	Insufficient details provided to evaluate	U	Insufficient details on cell counting methods			Yes		
Heeren	2018	30050535	Cohort baseline	U	Possible confounding by HIV status	L	All qualifying patients in range selected	U	Possible bias in selection of imaging areas			Yes		
Hellberg	2009	18976801	Cohort	L	Adjusted for clinical stage, other markers; HIV less of a concern in this time range	U	Insufficient details provided about selection to determine likelihood of bias	L	Pathologist blinded to clinical details	10	Minimum		Yes	
Hilders	1993	8264228	Cross-sectional	U	Difficult to assess with details provided	L	All patients with available tissue were selected; controls appropriate	U	Possible non-random areas assesed; also not stated whether cell counters were blinded			Yes		
Hirbod	2013	24006463	Cross-sectional	L	Controlled for or excluded based on potential confounders	L	Two appropriate control groups	L	Blinded assessment of tissue, full sections evaluated			Yes		
Hou	2012	22820395	Cross-sectional	L	Appropriate exclusions for potential confounders	L	Consecutive patients enrolled; normal control specimens from comparable population	U	"Randomly selected" high power fields possibly not truly random, not stated whether reviewer was blinded to outcome			Yes		
Hu	2015	25885042	Cross-sectional	L	Excluded based on likely confounders	L		U	"Randomly selected" areas may not be truly random			Yes		
Jaafar	2009	19808652	Cross-sectional	U	No details provided to evaluate	U	Insufficient details on patient selectino provided to determine	U	"Representative areas" selected for study and "randomly selected fields" possibly not truly random introduce possibility of bias			Yes		
Jordanova	2008	18381941	Cohort	U	No data on HIV status; did not control for cancer stage, type, etc.	L	All eligible cases in time range included	U	Cells counted in "ramdomly selected" fields likely not truly chosen at random; automated cell counting	5	Maximum	Yes	Yes	
Karageorgopoulou	2017	28659181	Clinical trial	H	Not controlled for prior chemotherapy, HIV status, primary vs recurrant cancer	U	Possible selection bias into randomized trial from which cases were drawn	L	Path reviewers blinded to clinical characteristics	0.02-6.75	Range		Yes	
Kobayashi	2004	15374995	Cross-sectional	H	Comparing HIV+ and HIV- patients from three different studies makes unmeasured confounding likely	U	Hospital controls used as normal tissue source, may not be representative	U	Not indicated whether cell counting was blind and fields not selected at random			Yes		
Kuppers	1998	25951354	Cross-sectional	H	No details provided; likely confounding by HIV status or other unconsidered factors	U	Insufficient details provided to evaluate	L	Blinded investigators, areas to evaluate selected randomly			Yes		
Li	2014	25423704	Cross-sectional	U	Insufficient details to evaluate	U	Insufficient details provided to evaluate; use of hospital controls for normal tissue	U	No stated whether cell counters were blinded; sections not selected randomly			Yes		
Liang	2018	30474571	RCT baseline	L		L	Consecutive patients recruited; patients likely representative of cancer patient population overall	H	T cells counted in fields with highest density, not randomly			Yes		
Loddenkemper	2009	19514119	Cross-sectional	L		L	Random selection of archived tissues	L	Random selection of fields to count, counters blinded to outcomes			Yes		
Lucena	2016	26545568	Cross-sectional	L	Stratified by HIV status, controlled for other potential confounders	U	Insufficient details to evaluate	L	Scoring system subjective but examiner blinded so any bias not likely differential			Yes		
Maldonado	2014	24477000	Clinical trial baseline	L	Excluded based on likely confounders	L		U	Regions of interest defined subjectively			Yes		
Maluf	2008	18343936	Cohort	L	Possible HIV confounding but unlikely given time period	H	Several exclusion reasons likely associated with T cell counts (surgical margins requiring hysterectomy, lesions too small for IHC)	U	Not stated whether cell counting was performed in a blind manner	4	Minimum	Yes	Yes	
Monnier-Benoit	2006	16427684	Cohort baseline	L	All patients immunocompetent	L	Controls from same cohort as CIN cases; cancers separate which is not ideal but unavoidable	U	Unclear whether cell counters were blinded			Yes		
Munk	2012	23017821	Cohort baseline	L	Excluded based on likely confounders	L	All eligibile patients in range asked to participate	U	Cell counting possibly not at random, difficult to ascertain			Yes		
Nakamura	2007	17433037	Cross-sectional	U	No details provided on probably confounders	U	Insufficient details to evaluate	U	No indication that high power fields selected randomly or that cell counters were blinded			Yes		Yes
Nedergaard	2007	17940503	Cross-sectional	U	Possible HIV confounding	U	Probably selected all cancers that met inclusion criteria but didn't explicitly state this in methods	U	Fields of view selected randomly; can't tell if counting procedure introduced possible information bias			Yes		
Nedergaard	2007	18184401	Cross-sectional	U	Possible HIV confounding	L	Consecutive patients recruited; possible bias in that patients with less advanced cancer may be more likely not to have tumor tissue in archival blocks but our analysis did not distinguish between stages so likely not relevant here	L	Random selection of tissue blocks and areas within tissues, systematic cell counting protocols			Yes		
Nedergaard	2008	17945335	Cohort	U	Possible HIV confounding	L	Consecutive eligible patients included	U	Fields of view selected systematically, unclear whether reviewers were blinded	5	Exactly		Yes	
Olaitan	1996	8805867	Cross-sectional	L	Careful screening of participants for likely confounders	U	No details on HIV- controls (the group included in this analysis)	U	Unclear how randomly sections were chosen for counting or whether reviewers were blinded			Yes		
Origoni	2013	24455729	Cohort	L	Exclusions based on all likely confounding factors	L	Consecutive patients enrolled	L	Path reviewer blinded	2	Exactly	Yes	Yes	
Ovestad*	2010	20512116	Cross-sectional	U	No discussion of potential confounders, difficult to evaluate	U	Insufficient details provided to evaluate	H	Highly unrepresentative areas selected for counting					Yes
Ovestad	2011	21421698	Cohort	U	No discussion of potential confounding factors, difficult to evaluate bias likelihood	U	Insufficient details to determine likelihood of selection bias	H	Unclear whether reviewers were blinded; only most severely dysplastic area was evaluated	0.23	Median	Yes	Yes	Yes
Peghini	2012	22749886	Cross-sectional	L	Excluded for likely confounders	U	Unclear whether normal controls from same population as CIN/cancer patients	U	Unclear whether reviewers we blinded; subjective scoring system			Yes		Yes
Piersma	2007	17210718	Cross-sectional	U	Possible HIV confounding	U	Unclear whether normal controls from same population as cancer patients	U	Unclear whether reviewers blinded			Yes		

Poppe	1995	7890250	Cross-sectional	L	Excluded based on likely confounders	U	Hysterectomy patients for noncervical benign pathology as normal tissue source; may not be representative	L	Entire epithelium evaluated by blinded pathologist			Yes		
Prata	2015	26059395	Cross-sectional	U	No discussion of potential confounders	U	Convenience samples possibly not representative	L	Random, blinded selection of tissue areas to count			Yes		
Pudney	2005	16093359	Cross-sectional	U	Some "normal" patients had cervical inflammation; can't tell whether these were included in analytic population	U	Hysterectomy patients for noncervical benign pathology as normal tissue source; may not be representative	U	Unclear whether cell counters were blinded or regions selected randomly			Yes		
Punt	2015	25795131	Cohort	U	Possible HIV confounding, other unknown factors due to long time range	U	All cases in range included but 20 year span raises issues of changing clinical practices, populations over time	L	Automated cell counting	5	Maximum	Yes	Yes	
Qinfeng	2013	23510275	Clinical trial baseline	U	No discussion of potential confounders, difficult to evaluate	U	Seem to have selected all eligible patients but didn't state this explicitly	U	Five fields selected "randomly" possibly not truly random, also unclear if reviewers blinded to clinical characteristics			Yes		
Roncalli	1988	2448545	Cross-sectional	L	No likely confounders for this population (pre-widespread HIV)	L	No details provided on patient selection other than hysterectomy for non-cervical reasons; seems a reasonable cross-section	U	Sections counted possibly not representative, cell counters not blinded			Yes		
Rosini	1996	8760019	Cross-sectional	L	Matched on likely confounders; stratified by HIV status	U	No details provided on subject selection so impossible to evaluate	U	Not stated whether pathologists were blinded to HIV status or how fields were selected			Yes		
Saglam	2019	31274701	Cohort	U	No details about patients makes possible confounding impossible to ascertain	U	No details on patient selection	H	Random regions of interest selected "under the direction of a pathologist" likely not truly random, also tissue samples taken from most invasive portion of tumor	9.4	Mean		Yes	
Shah	2011	21200385	Cohort	U	Insufficient details to evaluate	U	Insufficient details to evaluate	L	Whole slides counted	5	Minimum	Yes	Yes	
Silva	2010	20613932	Cross-sectional	L	Excluded based on likely confounders	U	Probably a random selection of eligible cases but did not specify this	U	Nonrandom areas were counted; unclear whether reviewers were blinded; insufficient slides possibly not at random			Yes		
Srivani	2003	12801265	Cross-sectional	U	Insufficient details to evaluate	U	Insufficient details to evaluate	U	Nonrandom and probably non blinded cell counting			Yes		
Syrjanen	1985	3002294	Cohort	L	Dates reduce possibility of HIV confoudning	L	Prospective study	U	Cells counted not likely truly random, although cell counting was blinded	1.7	Mean		Yes	
Syrjanen	1987	3032634	Cohort	L	Dates reduce possibility of HIV confoudning	L	Prospective study	U	Cell counted not likely truly random	2.1	Mean		Yes	
Szarewski	2001	11281472	Cohort baseline	L	Excluded based on likely confounders	U	Unclear whether cohort representative of normal population	L	Blinded, systematic cell counting			Yes		
Trimble	2010	21037100	Cohort	L	Exclusions based on likely confounding factors	U	Prospective study; HPV16 only could have an unknown effect vs other HPV types	U	Unclear how regions of interest were selected, whether selectors were blinded to outcomes	0.29	Exactly		Yes	
Vayrynen	1985	2989155	Cohort	L	Potential HIV confounding but well done study, series in 1980 makes unlikely	U	Consecutive women enrolled; unclear enrollmet criteria	L	Cell counter blinded to specimen identity	1.3	Mean	Yes	Yes	
Viac	1990	2168858	Cross-sectional	U	Insufficient details to evaluate	U	Insufficient details to evaluate	U	No details on high power field selection			Yes		
Wang	2014	25446402	Cross-sectional	U	Insufficient details to evaluate	L	Seem to have selected all eligible patients; appropriate normal controls	U	No details on high power field selection or indication of whether cell counters were blinded			Yes		
White	1997	9138451		U	Insufficient details to evaluate; not confident that a rural population is sufficient to rule out HIV confounding	U	Elective hysterectomy patients; insufficient details provided to evaluate potential selection bias	U	No indication whether cell counters blinded or how areas selected for evaluation			Yes		
Woo	2008	19035938	Cohort	U	Potential HIV confounding	U	No details provided	L	Pathologist blinded to clinical information	1	Exactly	Yes	Yes	
Wu	2011	21930068		U	Insufficient details to evaluate	U	Insufficient details to evaluate	L	Systematic, random field selection and blinded reviewers					Yes

Table S2. Quality Review. A quality review was conducted for each of the studies included in the the quantitative meta-analysis, qualitative CD25 analysis, and/or longitudinal analysis to record the likelihood of confounding, selection bias, and information bias. Abbreviations: PMID, PubMed ID; NR, not reported; L, low; U; unknown; H, high.

Table S3. Sensitivity Analysis Results

A. Exclusion of cancer-adjacent normal, exclusion of unknown cancer type, or inclusion of all cancers (mean (95% CI))*

	Normal		LGCIN†	HGCIN†	Cancer		
	All†	Excluding cancer-adjacent			Squamous and unreported/ unknown†	Squamous only	All including known adenocarcinomas
Total							
CD3	341 (81, 601)	341 (81, 601)	164 (29, 298)	214 (77, 352)	712 (447, 977)	620 (342, 898)	638 (368, 908)
CD4	209 (44, 375)	209 (44, 375)	139 (7, 271)	143 (79, 206)	287 (191, 383)	305 (223, 387)	262 (172, 353)
CD8	127 (7, 248)	127 (7, 248)	141 (43, 238)	173 (120, 225)	552 (394, 710)	443 (312, 574)	498 (365, 631)
CD4:CD8 Ratio	0.93 (0.61, 1.24)	0.93 (0.61, 1.24)	0.75 (0.33, 1.18)	0.70 (0.51, 0.88)	0.80 (0.47, 1.13)	0.65 (0.46, 0.85)	0.76 (0.46, 1.06)
FoxP3	107 (-104, 318)	107 (-104, 318)	4 (1, 7)	52 (41, 63)	391 (282, 500)	183 (33, 332)	323 (235, 412)
Epithelial							
CD3	146 (104, 187)	149 (105, 193)	65 (36, 94)	137 (103, 170)	247 (178, 317)	383 (210, 557)	264 (196, 332)
CD4	94 (63, 125)	106 (65, 148)	19 (11, 27)	16 (6, 27)	52 (38, 67)	93 (28, 157)	52 (38, 67)
CD8	137 (76, 199)	143 (77, 209)	37 (19, 55)	46 (25, 66)	126 (97, 155)	223 (142, 305)	97 (75, 119)
CD4:CD8 Ratio	0.81 (0.53, 1.10)	0.89 (0.58, 1.19)	1.17 (0.71, 1.63)	0.75 (0.37, 1.12)	0.66 (0.42, 0.91)	0.46 (0.28, 0.64)	0.66 (0.42, 0.91)
FoxP3	19 (3, 36)	19 (3, 36)	0.4 (0.3, 0.4)‡	7 (0, 15)	8 (6, 10)	59 (32, 85)	11 (9, 13)
Stromal							
CD3	381 (130, 632)	397 (109, 685)	303 (193, 413)	458 (358, 557)	838 (560, 1117)	954 (492, 1415)	1029 (738, 1320)
CD4	149 (52, 246)	273 (169, 378)	142 (94, 190)	60 (27, 92)	185 (122, 249)	187 (88, 286)	185 (122, 249)
CD8	247 (136, 358)	293 (246, 341)	157 (50, 264)	174 (108, 240)	395 (274, 517)	448 (286, 610)	395 (274, 517)
CD4:CD8 Ratio	0.68 (0.51, 0.85)	0.75 (0.66, 0.84)	0.92 (0.54, 1.30)	1.50 (0.98, 2.03)	0.90 (0.60, 1.20)	1.00 (0.50, 1.51)	0.90 (0.60, 1.20)
FoxP3	--	--	7 (6, 8)	9 (3, 15)	56 (45, 67)	103 (-9, 216)	56 (45, 67)

B. Stratification by quantification metric, cells per unit area or cells per HPF (mean (95% CI))*

	Normal	LGCIN	HGCIN	Cancer
**Cells per unit area**				
Total				
CD3	28 (24, 32)‡	--	441 (424, 458)‡	699 (685, 713)‡
CD4	12 (9, 15)‡	--	206 (193, 219)‡	312 (301, 323)‡
CD8	16 (13, 19)‡	--	235 (224, 246)‡	387 (379, 395)‡
CD4:CD8 Ratio	0.75 (0.53, 0.97)‡	--	0.88 (0.81, 0.95)‡	0.81 (0.77, 0.84)‡
FoxP3	0.1 (-0.1, 0.4)‡	2 (0, 5)	9 (5, 12)	59 (38, 81)
Epithelial				
CD3	232 (131, 334)	65 (29, 101)	132 (89, 175)	283 (189, 376)
CD4	86 (53, 119)	22 (3, 41)	8 (2, 13)	49 (28, 70)
CD8	132 (64, 201)	36 (13, 59)	35 (10, 61)	135 (100, 170)
CD4:CD8 Ratio	0.81 (0.49, 1.13)	0.57 (0.27, 0.87)	0.54 (0.15, 0.94)	0.55 (0.27, 0.83)
FoxP3	--	0.4 (0.3, 0.4)‡	2.1 (1.9, 2.2)	5.6 (3.6, 7.6)
Stromal				
CD3	455 (333, 577)	388 (161, 615)	627 (448, 806)	1170 (366, 1973)
CD4	149 (52, 246)	155 (102, 209)	29 (9, 50)	302 (105, 499)
CD8	247 (136, 358)	178 (57, 300)	196 (93, 299)	630 (339, 920)
CD4:CD8 Ratio	0.68 (0.51, 0.85)	0.92 (0.54, 1.30)	1.54 (0.96, 2.12)	0.65 (0.33, 0.96)
FoxP3	--	6.8 (5.6, 7.9)‡	6.6 (-0.3, 13.4)	20.8 (11.8, 29.7)
**Cells per HPF**				
Total				
CD3	508 (-183, 1199)	164 (29, 298)	161 (98, 223)	713 (431, 996)
CD4	323 (-119, 765)	139 (7, 271)	129 (52, 207)	282 (180, 385)
CD8	184 (-65, 433)	141 (43, 238)	160 (108, 211)	586 (299, 872)
CD4:CD8 Ratio	1.04 (0.56, 1.53)	0.75 (0.33, 1.18)	0.66 (0.46, 0.86)	0.85 (0.46, 1.24)
FoxP3	216 (174, 257)‡	53 (35, 70)‡	302 (15, 589)	589 (140, 1037)
Epithelial				
CD3	29 (18, 40)	66 (53, 80)‡	144 (63, 225)	202 (50, 354)
CD4	116 (91, 140)	16 (15, 17)‡	56 (-27, 140)	79 (24, 135)
CD8	155 (96, 215)	42 (33, 51)‡	65 (10, 119)	115 (17, 213)
CD4:CD8 Ratio	0.84 (0.61, 1.07)	4.30 (3.53, 5.07)‡	2.23 (-0.24, 4.69)	0.80 (0.59, 1.01)
FoxP3	19 (3, 36)	--	12 (0, 23)	18 (9, 26)
Stromal				
CD3	15 (8, 23)‡	51 (44, 58)‡	384 (137, 631)	259 (134, 384)
CD4	--	86 (31, 141)‡	203 (-108, 514)	116 (55, 178)
CD8	--	64 (-97, 225)‡	180 (-19, 379)	117 (51, 182)

CD4:CD8 Ratio	--	--	1.34 (0.57, 2.10)‡	1.27 (0.97, 1.56)
FoxP3	--	--	45 (-10, 99)	82 (38, 126)

C. Restriction to explicitly reported values for CD3, CD4, CD8 and the CD4:CD8 ratio (mean (95% CI))*

	Normal	LGCIN	HGCIN	Cancer
Total				
CD3	--	76 (60, 93)	93 (59, 128)	147 (115, 178)
CD4	209 (44, 375)	199 (165, 232)	196 (161, 230)	324 (224, 425)
CD8	127 (7, 248)	141 (43, 238)	173 (120, 225)	552 (394, 710)
CD4:CD8 Ratio	0.86 (0.38, 1.34)	0.42 (0.31, 0.53)‡	0.50 (0.41, 0.60)	0.11 (0.02, 0.20)‡
Epithelial				
CD3	59 (31, 87)	74 (57, 91)	210 (127, 293)	554 (322, 786)
CD4	94 (63, 125)	19 (11, 27)	16 (6, 27)	52 (38, 67)
CD8	138 (103, 174)	37 (19, 55)	46 (25, 66)	166 (117, 215)
CD4:CD8 Ratio	1.80 (-0.94, 4.53)	1.17 (0.71, 1.63)	0.79 (0.31, 1.27)	0.98 (0.50, 1.46)
Stromal				
CD3	283 (29, 536)	153 (-50, 356)	619 (349, 890)	1450 (543, 2356)
CD4	84 (52, 117)	142 (94, 190)	60 (27, 92)	185 (122, 249)
CD8	247 (136, 358)	157 (50, 264)	174 (108, 240)	395 (274, 517)
CD4:CD8 Ratio	0.75 (0.66, 0.85)‡	0.92 (0.54, 1.30)	1.61 (0.95, 2.27)	1.16 (0.44, 1.89)

* All results are in cells/mm^2

† Reported in main manuscript

‡ Categories with a single study. Narrow CI should not be interpreted as high

-- Categories with no studies

Table S3. Sensitivity Analysis Results. Meta-analysis results including means and 95% confidence intervals for the following sensitivity analyses: A. exclusion of cancer-adjacent normal, exclusion of unknown cancer type, and inclusion of all cancers B. stratification by quantification metric, cells/mm^2 or cells per high power field (HPF), and C. restriction to explicitly reported values for CD3, CD4, CD8, and the CD4:CD8 ratio. Abbreviations: Abbreviations: HPF, high power field; CI, confidence interval; LG, low-grade; CIN, cervical intraepithelial neoplasia; HG, high-grade

Table S4. Studies Included in CD25 Analysis

			Disease level					Tissue type		
**First Author**	**Year**	**PMID**	**Normal**	**LGCIN**	**HGCIN**	**Cancer**	**Other**	**Epithelial**	**Stromal**	**Total**
Adurthi	2008	18593438	37		30	30	37			X
Bedoya	2013	22290207		26	46	24		X	X	
Ferrandina	2006	16609015				27			X	
Goncalves	2009	19689792	4	13	30			X	X	
Nakamura	2007	17433037	24		31	28	13	X	X	
Ovestad*	2010	20512116			55			X	X	
Ovestad*	2011	21421698			55			X	X	
Peghini	2012	22749886		21	34	8				
Wu	2011	21930068				10	8	X		

* These are the same 55 cases reported twice in the literature

Table S4. Studies Included in CD25 Analysis. Studies included in the CD25 analysis are listed, including record identification information and an indication of sample size at each disease stage as well as which tissues types were measured. Not all studies included quantified results for CD25. Abbreviations: PMID, PubMed identification number; LG, low grade; CIN, cervical intraepithelial neoplasia; HG, high grade.
